# Spatially Fractionated Radiation Therapy for Palliation in Patients With Large Cancers: A Retrospective Study

**DOI:** 10.1016/j.adro.2024.101665

**Published:** 2024-10-31

**Authors:** Federico Iori, Valeria Trojani, Alice Zamagni, Patrizia Ciammella, Mauro Iori, Andrea Botti, Cinzia Iotti

**Affiliations:** a Radiation Oncology Unit, Azienda USL-IRCCS di Reggio Emilia, Reggio Emilia, Italy; b Clinical and Experimental Medicine PhD Program, Department of Biomedical, Metabolic, and Neural Sciences, University of Modena and Reggio Emilia, Modena, Italy; c Medical Physics Unit, Azienda USL-IRCCS di Reggio Emilia, Reggio Emilia, Italy

## Abstract

**Purpose:**

Spatially fractionated radiation therapy (SFRT) is an irradiation technique developed to improve large cancer response. Although preliminary studies report highly positive results, data are still limited. The aim of this retrospective monocentric study was to investigate SFRT safety and activity.

**Methods and Materials:**

We analyzed all patients who underwent SFRT as a palliative treatment for large solid extracranial cancer (>4.5 cm) at our institution. The primary endpoint was objective response rate assessment at 3 months. Additionally, patients’ antalgic response, target volume reduction, and performance status modification were measured. Toxicity data were recorded.

**Results:**

From November 2021 to August 2023, 20 consecutive patients (20 lesions) underwent SFRT. We prescribed a minimum dose of 20 Gy in 5 fractions to 95% of the Planning Target Volume (PTV_20) and a minimum dose of 50 Gy to 50% of the sphere volume. The median beam-on time was 5 minutes (IQR_1-3_, 4-7 minutes; range, 3-16 minutes). Patients’ median age was 70 years (range, 18-85 years). The median lesion volume was 560.4 cm3 (IQR_1-3_, 297.4-931.5 cc; range, 168.3-3838.3 cm3). Of the 20 patients, 14 and 10 were alive at 3 and 6 months, respectively. The 3-month objective response rate was 79% (95% CI, 49%-95%), with a median target volume reduction of 54% (IQR_1-3_, 32%-69%; range, 6%-80%). At 6 months, all patients were free from local disease progression. All patients reported an antalgic response with a rapid onset. All treatment-related toxicities occurred within 1 month after SFRT and quickly recovered. No acute toxicity ≥ grade 3 and late toxicity was reported. No patient experienced a worsening in performance status.

**Conclusions:**

Our results provide further evidence supporting SFRT as a safe and promising option for palliative patients affected by large neoplastic lesions.

## Graphical abstract

**Figure:**
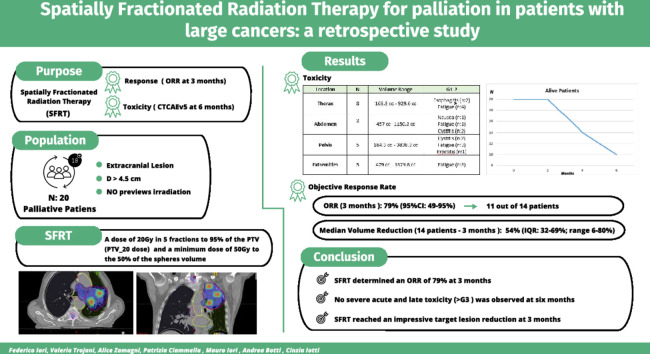

## Introduction

The management of large and unresectable tumors represents an open and concerning issue in the palliative setting. Large lesions can determine organ compression and invasion, with significant symptoms that impair patients’ quality of life. Moreover, patients undergoing palliative treatment are often compromised because of advanced disease and previous oncologic treatments. Consequently, these patients do require prompt management that carefully considers the tradeoff between treatment efficacy and side effects.^1^

Systemic therapy has poor efficacy on large neoplastic lesions and can be excessively toxic.^2^ Surgery is generally excluded, because removing large lesions requires risky operations, which is unacceptable in a palliative setting.^3^

Hence, palliative radiation therapy (p-RT) is usually the preferred option to achieve symptom control and delay local progression. Standard p-RT is performed with homogeneous low-dose regimens, irrespectively from tumor histology.^4^^,^^5^ Considering that treatment priority is tolerance over efficacy, p-RT is unable to obtain a significant and durable local control, especially for massive tumor volumes. Spatially fractionated radiation therapy (SFRT) can overcome this issue.^6^ SFRT is a technique that delivers an intentional nonuniform dose to the target volume, with some areas receiving higher doses with a simultaneous integrated boost. The hotspots are located inside the target and distant from the surrounding organs at risk.^7^ This can improve tumor response, minimizing treatment-related toxicity. Published clinical experiences reported very encouraging results; however, the studies are small, retrospective, and heterogeneous.^8^^,^^9^

The aim of this retrospective work was to provide further data on palliative SFRT in a cohort of patients with large neoplastic lesions.

## Methods and Materials

### Study population

This study is part of a retrospective work on patients who underwent palliative irradiation at our institution. The study was approved and authorized by the ethical committee and by the internal review board. In the present report, we examined only the patients who underwent SFRT. All the patients were aged ≥18 years, not previously irradiated on the same region, and with a nonbrain large tumor (diameter ≥4.5 cm) unresponsive to systemic therapy and/or not amenable to surgery or chemotherapy.

### Study outcomes

The objective of this study was to assess the activity and toxicity of SFRT in patients with bulky tumors addressed for p-RT. The objective response rate (ORR) of the irradiated lesions was evaluated at 3 months, according to the Response Evaluation Criteria in Solid Tumors (RECIST) 1.1.. Treatment-related toxicity was assessed using the Common Terminology Criteria for Adverse Events, version 5. Additionally, the 3- and 6-month volume modifications were analyzed. Pain response and Eastern Cooperative Oncology Group (ECOG) performance status (PS) were also evaluated at 3 and 6 months.

### Statistical analysis

Patients and treatment data were summarized with descriptive statistics (median, ranges, and the first and third IQRs [IQR_1-3_]). The Clopper-Pearson exact method formula was used to calculate the ORR CI at a 95% confidence level. All statistical analysis was performed with JupiterNotebook_Python _v3.12.0.

## Results

### Patients description

From November 2021 to August 2023, 20 patients (20 target lesions) underwent a 5-fraction SFRT treatment and were included in this study. The SFRT was the only radiation treatment delivered to the lesion. No concurrent antineoplastic therapy was administered during SFRT, with a 2-week washout for all patients. The median lesion diameter was 13 cm (IQR_1-3_, 9.5-17 cm; range, 7.5-26 cm), with a median volume of 560.4 cm3 (IQR_1-3_, 297.4-931.5 cm3; range, 168.3-3838.3 cm3). The patients’ median age was 70 years (IQR_1-3_, 64-72 years; range, 19-84 years). The median ECOG PS was 2 (IQR_1-3_, 1-2; range, 0-3), with 60% of patients being hospitalized in the month prior to SFRT delivery. The median Numeric Pain Rating Scale was 6 (IQR_1-3_, 5-7; range, 0-10;), with 85% of patients receiving antalgic therapy. Six patients were treated for the primary tumor and 14 for secondary lesions. Target lesions were progressing after 1-6 lines of systemic therapy, except for 3 chemotherapy-naïve patients. Patients’ and lesions’ characteristics are shown in Table 1 (details in Appendix E1 Supplementary Material*).*

**Table 1:** Patients’ and lesions’ characteristics

Patients (N = 20)	
Age (y), median (range)	70 (19-84)
Sex	Males: 10
	Females: 10
BMI, median (range)	26.3 (20-34.7)
ECOG PS	PS 0: 1
	PS 1: 7
	PS 2: 8
	PS 3: 4
NPRS*	No pain: 1
	Mild pain: 3
	Moderate pain: 9
	Severe pain: 7
Systemic therapies	Naïve: 3
	I line: 5
	II lines: 4
	III lines: 6
	IV lines: 2
Recent hospitalization†	Yes: 12
	No: 8
Target	Primary tumor: 6
	Metastasis: 14
Primary	Lung cancer: 6
	Renal cancer:3
	Ovarian cancer: 3
	Skin melanoma: 2
	Sarcoma: 2
	Prostate cancer: 1
	Bladder cancer: 1
	Pleural mesothelioma: 1
	Colorectal cancer: 1
Comorbidity	Hypertension: 10
	COPD: 3
	heart disease: 6
	Diabetes: 5
	Dyslipidemia: 4
	CKD: 2
	CVD: 2

*Abbreviations:* BMI = body mass index; CKD = chronic kidney disease; COPD = chronic obstructive pulmonary disease; CVD = cerebrovascular disease; ECOG PS: Eastern Cooperative Oncology Group Performance Status; NPRS = Numeric Pain Rating Scale.

⁎ No pain (NPRS: 0), mild pain (NPRS: 1-3), moderate pain (NPRS: 4-6), and severe pain (NPRS: 7-10).

† Hospitalized patient or hospitalization in the month before spatially fractionated radiation therapy.

### SFRT planning and delivery

All patients underwent a planning computed tomography with or without contrast enhancement. The referent physician contoured the gross tumor volume (GTV). A margin of 5-8 mm was added to the GTV to create the Planning Target Volume (PTV). Then, a series of "spheres” with diameters of 1-1.5 cm were delineated and regularly placed inside the GTV. Their center-to-center distance ranged between 2.5 and 4.0 cm. The minimum distance between the spheres’ edge and the adjacent organs at risk was 1 cm. The median sphere number was 8 (IQR_1-3_, 6-13; range, 4-17). We prescribed a minimum dose of 20 Gy in 5 fractions to 95% of the PTV (PTV_20) and a minimum dose of 50 Gy to 50% of the sphere volume; see Table 2 for the prescription details.

**Table 2:** In the table, the prescriptions of each patient and the main plan dose metrics are highlighted.

Patients	PTV_20				Spheres			
	Prescription	Dnear_min (Gy)	D50 (Gy)	Volume (cm3)	Dnear_min (Gy)	D50 (Gy)	Dnear_max (Gy)	Volume (cm3)
P_1	5 × 4 Gy	19.6	25.7	1570.0	47.2	50.8	54.1	26.5
P_2	5 × 5 Gy	24.3	28.3	378.5	47.5	50.2	52.8	6.0
P_3	5 × 5 Gy	24.5	28.6	1179.2	54.3	56.8	58.4	9.3
P_4	5 × 5 Gy	24.4	30.0	4320.3	54.8	59.5	63.2	17.9
P_5	5 × 4 Gy	19.3	26.3	2187.0	55.2	60.0	66.0	20.1
P_6	5 × 5 Gy	24.2	29.4	1264.3	51.7	55.5	59.5	15.8
P_7	5 × 4 Gy	19.3	24.0	1755.5	45.7	50.2	53.6	19.2
P_8	5 × 5 Gy	24.6	28.4	483.6	49.0	53.8	57.6	10.1
P_9	5 × 5 Gy	24.6	28.4	251.7	54.3	57.7	59.7	2.1
P_10	5 × 5 Gy	24.6	29.5	258.2	52.6	54.8	56.1	2.4
P_11	5 × 4 Gy	19.0	25.3	666.7	46.5	50.1	53.8	8.1
P_12	5 × 4 Gy	19.6	25.4	733.4	48.9	51.2	52.7	11.3
P_13	5 × 4 Gy	19.5	25.8	1329.9	47.8	50.8	53.4	21.7
P_14	5 × 4 Gy	19.6	24.3	480.8	49.0	51.9	53.9	5.4
P_15	5 × 4 Gy	19.5	22.7	859.3	46.8	50.0	52.3	5.8
P_16	5 × 5 Gy	24.5	28.7	554.2	55.8	59.0	61.4	6.7
P_17	5 × 4 Gy	19.5	25.6	1685.1	50.0	52.4	54.4	21.8
P_18	5 × 5 Gy	24.3	30.6	1194.2	50.9	56.3	61.1	12.1
P_19	5 × 4 Gy	19.6	23.0	561.4	48.8	51.4	53.6	4.0
P_20	5 × 5 Gy	24.3	30.7	888.2	52.6	56.2	59.4	15.4

*Abbreviations:* PTV_20: planning target volume that receive at least 20 Gy in 5 fractions to 95% of the PTV_20; Dnear_min: minimal receivedmdose by the 98% of PTV_20 volume; Dnear_max: maximal received dose by the 2% of PTV_20 volume; D50: dose received at least by the 50% of the PTV_20.

**Figure 1:**
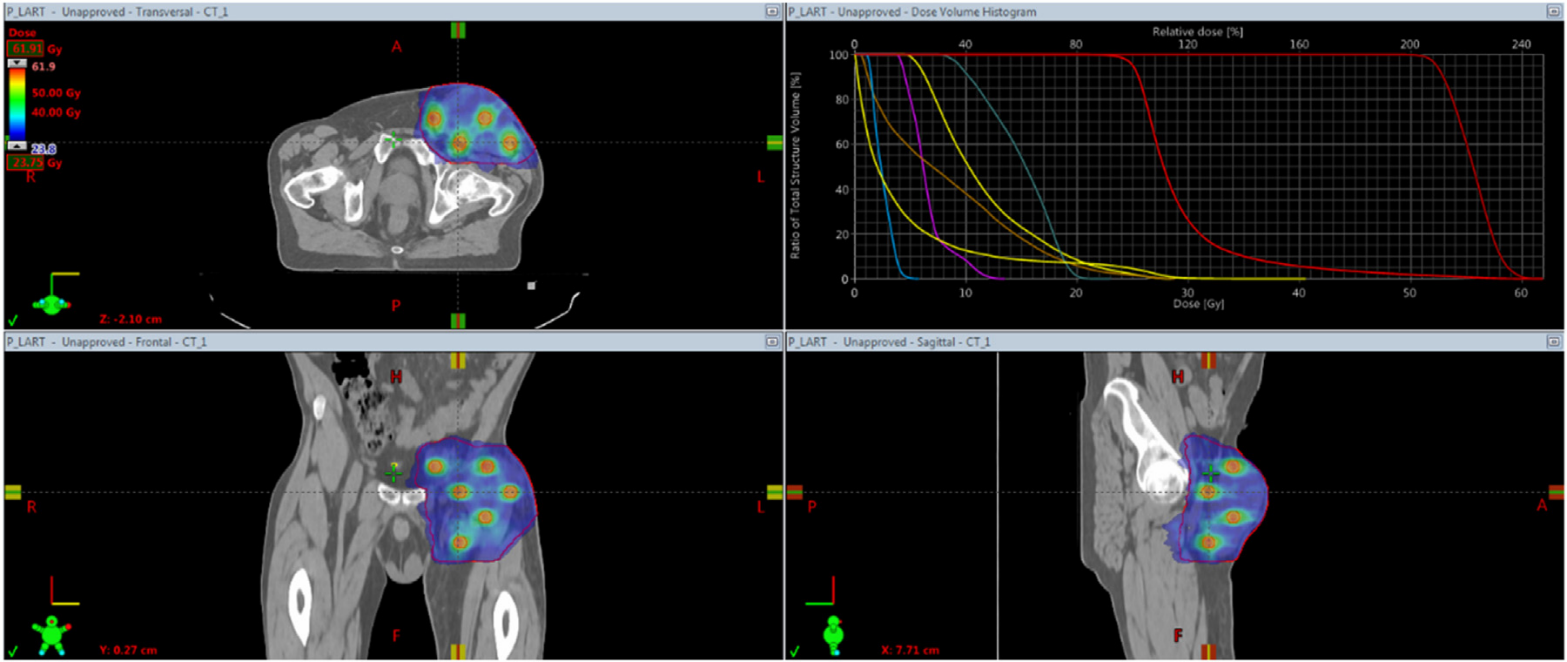
The figure shows the sphere disposition in the target lesion of patient_6 and its associated Dose Volume Histogram (DVH). Details of all patients are in the Supplementary Material Appendix E1.

The Eclipse Treatment Planning System (Varian) was employed to generate volumetric modulated arc therapy plans. The SFRT was delivered with a TrueBeam-STx (Varian), with a median number of 6 arcs (IQR_1-3_, 6-13; range, 4-8 arcs). The patient set-up was verified with a daily online volumetric image guided radiation therapy. All plans were verified with delivery quality assurance with the PTW Octavius 4D phantom and the 1000 SRS detector and analyzed using Verisoft v 8.1 (PTW). Local gamma analyses were used to compare the measured doses by the Treatment Planning System, with 2 mm/3% metrics and a 10% dose threshold. We set the gamma passing rate acceptability threshold to 90%. The median beam-on time was 5 minutes (IQR_1-3_, 4-7 minutes, range, 3-16 minutes). All patients successfully completed their 5-fractions treatment. Further details are in the Appendix E1. Supplementary Material An example of SFRT treatment is reported in Fig. 1.

### Outcomes

Fourteen (70%) and 10 (50%) patients were alive at 3 and 6 months, respectively. The 3-month ORR was 79% (95% CI, 49%-95%). The median target volume reduction was 54% (IQR_1-3_, 32%-69%; range, 6%-80%), with 11 and 3 patients experiencing partial response and stable disease, respectively. At 6 months, the response was maintained or improved in 9 out of 10 patients, with a median target volume reduction of 71% (IQR_1-3_, 39%-77%; range, 14%-93%). At 6 months, all patients were free from local disease progression.

A complete pain response was observed in 57% (8/14) and 70% (7/10) of patients at 3 and 6 months, respectively. All other patients had a partial antalgic response (≥2 points reduction from the baseline Numeric Pain Rating Scale). Interestingly, all patients reported a rapid onset of the antalgic response. At 3 months, ECOG PS was improved in 57% (8/14) of the patients and stable in 43% (6/14). A similar trend was observed at 6 months, with 70% (7/10) and 30% (3/10) of patients showing improved or stable PS, respectively. Grade 1-2 acute toxicity was observed in 13 out of 20 patients. Fatigue was the most commonly reported symptom (11/20). All treatment-related toxicities occurred within 1 month after SFRT and quickly recovered. No acute toxicity ≥ grade 3 and late toxicity was reported. In all patients, death that occurred was caused by multiorgan failure consequent to disease progression, except for 1 lung-irradiated patient who died because of sepsis originating from diverticulitis.

## Discussion

In this retrospective study, we analyzed the outcomes of patients treated with palliative SFRT for large tumors using a 5-day fractionation schema. We observed an ORR of 79% at 3 months, with a reduction of tumor volume up to 80%. In responsive patients, the response was maintained or improved through time. Only 1 patient with a dramatic response at 3 months had a local regrowth at 6 months but did not fulfill the RECIST 1.1 criteria for progression. At 6 months, all patients were in local control. Complete pain response was reported in 57% and 70% of patients at 3 and 6 months, respectively. Notably, the SFRT treatment determined a significant and prompt antalgic benefit in all treated patients, irrespective of the lesion response. Although 60% of the patients had a baseline PS ≥ 2, the treatment was well tolerated. No grade ≥3 acute toxicity or any grade late toxicity was observed, as well as no PS deterioration.

Recently, several clinical experiences with SFRT reported notable responses, with substantial tumor volume reduction and acceptable toxicity.^8^^,^^9^ However, the data are difficult to interpret because different strategies have been used. In a cohort of 30 metastatic patients with large tumors (>5 cm), Ferini et al^10^ investigated the clinical response and toxicity of a positron emission tomography-guided SFRT followed by homogeneous irradiation of the entire GTV. The overall clinical response was 89%, with an antalgic benefit in all irradiated patients.^10^ Similar positive outcomes were reported by Ahmed et al.^11^ They treated 53 patients with metastatic or unresectable large sarcoma with SFRT treatment followed by a consolidative external beam RT.^11^ A different and shorter SFRT approach was investigated in the phase 1 LITE SABR M1 trial, where 20 patients with large cancers (diameter >4.5 cm) were treated in 5 fractions of SFRT without severe toxicity.^7^

Our study has some limitations. It is a retrospective study with a limited number of heterogeneous patients, with only half surviving at 6 months. In addition, no patient-reported outcome measures were collected despite their importance in a palliative setting. A strength of the study is that all patients were treated with the same technique, fraction schema, and a pretty homogeneous dose prescription.

## Conclusions

In conclusion, our results suggest that in large solid cancers, SFRT is able to determine an impressive response with a low toxicity profile, even in compromised or fragile patients. Considering its potential role in the palliative setting, this technique needs to be further investigated in prospective trials.

## Disclosures

None.

## Appendix Supplementary materials

Supplementary_Material Image, application 1

## Footnotes

Supplementary material associated with this article can be found in the online version at doi:10.1016/j.adro.2024.101665.
