# Supplementary material for: Spatially Fractionated Radiation Therapy for Palliation in Patients With Large Cancers: A Retrospective Study
Source: Adv Radiat Oncol. 2024 Oct 31;10(1):101665. doi: 10.1016/j.adro.2024.101665 (PMC11647081; doi:10.1016/j.adro.2024.101665)
Supplement: Supplementary_Material [file mmc1.docx]

| Appendix_E |
| --- |

*In this Appendix_E, we report how we calculated the Objective Response Rate in the study population. In addition, we show the details of each patient who underwent Spatially Fractionated Radiation Therapy (SFRT). For each patient, a screenshot of the delivered plans was dysplayed with the 20Gy isodose line.*

**OBJECTIVE RESPONSE RATE**

$$ORR=\frac{Complete Responses+Partial Responses}{Number of patients}$$

According to the National Cancer Institute (NCI) the ORR is defined as: “*The percentage of people in a study or treatment group who have a partial response or complete response to the treatment within a certain period of time. A partial response is a decrease in the size of a tumor or in the amount of cancer in the body, and a complete response is the disappearance of all signs of cancer in the body. In a clinical trial, measuring the objective response rate is one way to see how well a new treatment works. Also called ORR*.”

| RECIST1.1* | |
| --- | --- |
| Complete Response (CR) | target lesion disapperence |
| Partial Response (PR) | ≥ 30% target lesion reduction |
| Stable Disease (SD) | No PR or SD |
| Progressive Disease (PD) | ≥ 20% target lesion increase |

** As SFRT is currently considered a local treatment, we will use the RECIST1.1 criteria only considering the target lesion and not the outfield metastatic burden, to assess lesions response.*

Please, see the reference link below for further information.

<https://www.cancer.gov/publications/dictionaries/cancer-terms/def/objective-response-rate>

**Constraints Adopted for Parallel and Serial Organs**

| **Serial Tissue** | | **Constrains** | |
| --- | --- | --- | --- |
| **Bladder** | | D 0.035 cc < 38 Gy | |
| **Bowel** | | D 0.035 cc < 38 Gy | |
| **Brachial plexus (R & L)** | | D 0.035 cc < 32.5 Gy | |
| **Bronchial_Tree** | | D 0.035 cc < 35 Gy | |
| **Duodenum** | | D 0.035 cc < 35 Gy | |
| **Esophagus** | | D 0.035 cc < 35 Gy | |
| **Femur Head (R & L)** | | D 10.0 cc < 30 Gy | |
| **Great Vessels** | | D 0.035 cc < 53 Gy | |
| **Heart** | | D 0.035 cc < 38 Gy | |
| **PRV_Brainstem** | | D 0.035 cc < 28 Gy | |
| **PRV_Cauda Equina** | | D 0.035 cc < 31.5 Gy | |
| **PRV_Spinal Cord** | | D 0.035 cc < 28 Gy | |
| **Rectum** | | D 0.035 cc < 38 Gy | |
| **Stomach** | | D 0.035 cc < 32 Gy | |
| **Skin** | | D 0.035 cc < 38.5 Gy | |
| **Parallel Tissue** | **Critical Dose** | | **Critical Volume** |
| **Lungs - GTV** | 12.5 Gy | | <1500 cc |
|  | 13.5 Gy | | < 1000 cc |
|  |  |  | < 37% |
| **Liver - GTV** | 21 Gy | | <700 cc |
| **Renal cortex (bilateral) - GTV** | 28 Gy | | < 200 cc |

**SFRT PATIENTS**

| PATIENT_01 |
| --- |


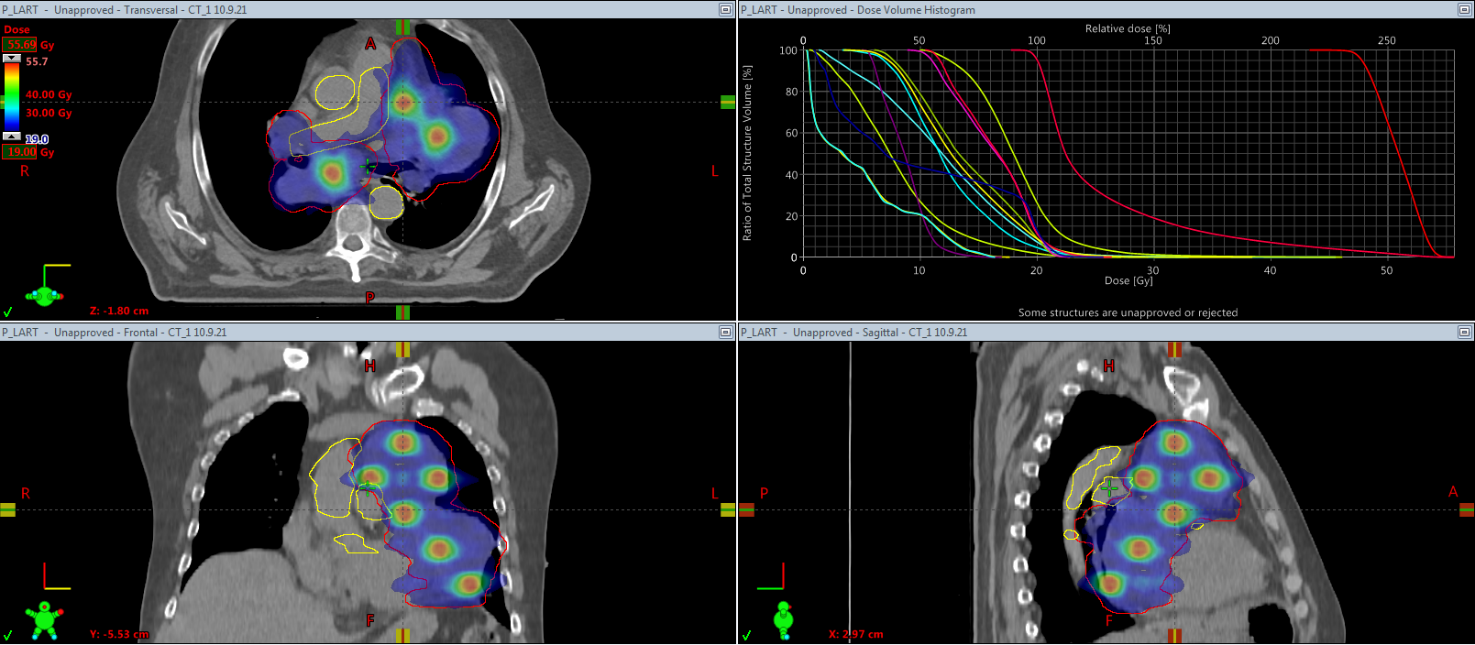


| DEMOGRAPHIC | | | |
| --- | --- | --- | --- |
| Age | | 70 | |
| Sex | | Male | |
| BMI | | 34.58 (105 kg – 174 cm ) | |
| CLINICAL DATA | | | |
| ECOG PS | | 3 | |
| NPRS | | 5 | |
| Primary | | Sarcomatoid lung cancer | |
| Tumor max diameter | | 19 cm | |
| Tumor location | | Thorax | |
| Stage | | IV | |
| Target lesion | | Primary tumor | |
| Antalgic Therapy | | Yes | |
| Therapy Lines | | I line: Carboplatin-Paclitaxel  II line: Nivolumab  III linea: Vinorelbine | |
| Current Systemic therapy | | Vinorelbine | |
| Hospitalization in the previews month | | No | |
| Comorbidity | | Bilateral inguinal hernia  Seventh (7th) nerve palsy  Superficial venous thrombosis | |
| SFRT DETAIL | | | |
| GTV dimension | | 946 cc | |
| PTV dimension | | 1570 cc | |
| Vertices_number | | 17 | |
| Energy | | Photon 6 MV FFF | |
| Arcs number | | 7 | |
| Monitor Units | | 5359.3 | |
| Beam on time | | 4.59 min | |
| SFRT RELATED TOXICIY | | | |
| G1 | | 0 | |
| G2 | | Esophagitis | |
| G3 | | 0 | |
| G4 | | 0 | |
| FOLLOW UP | | | |
|  | 3 months | | 6 months |
| Tumor volume | 383.2 cc | | 269.7 cc |
| NPRS | 2 | | 2 |
| ECOG PS | 1 | | 1 |
|  | | | |

| **PATIENT_02** |
| --- |


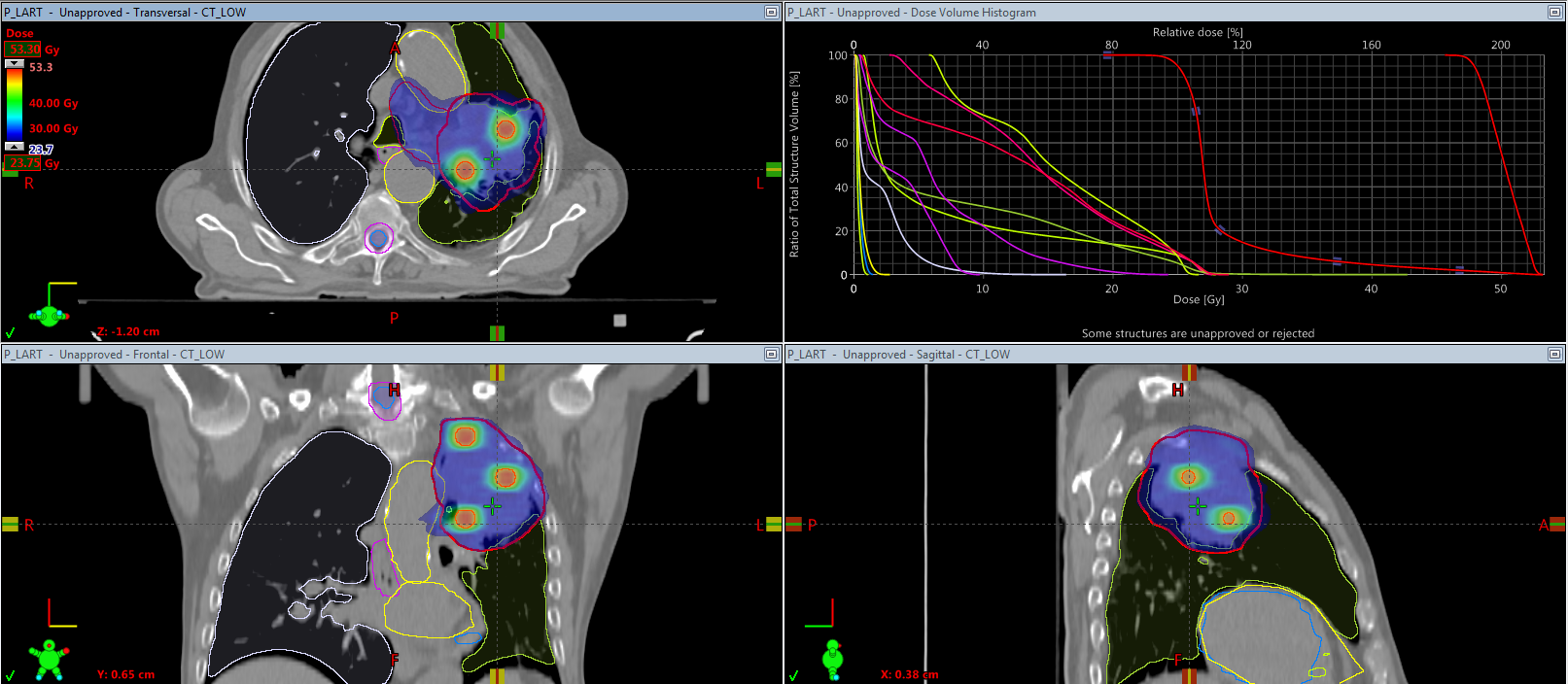


| DEMOGRAPHIC | | | |
| --- | --- | --- | --- |
| Age | | 84 | |
| Sex | | Male | |
| BMI | | 21.60 (56 kg – 161 cm) | |
| CLINICAL DATA | | | |
| ECOG PS | | 1 | |
| NPRS | | 5 | |
| Tumor max diameter | | 9 cm | |
| Primary | | NSCLC | |
| Target location | | Thorax | |
| Stage | | Locally Advanced | |
| Target lesion | | Primary tumor | |
| Antalgic Therapy | | No | |
| Therapy Lines | | No | |
| Current Systemic oncological therapies | | No | |
| Hospitalization in the previews month | | No | |
| Comorbidity | | Hyschemic heart disease  COPD  Dyslipidemia | |
| SFRT_DETAIL | | | |
| GTV dimension | | 223 cc | |
| PTV dimension | | 378.5 cc | |
| Vertices_number | | 4 | |
| Monitor Units | | 2635 | |
| Energy | | Photon 6 FFF | |
| Arcs | | 6 | |
| Beam on time | | 2.26 min | |
| SFRT_RELATED_TOXICIY | | | |
| G1 | | Fatigue | |
| G2 | | 0 | |
| G3 | | 0 | |
| G4 | | 0 | |
| Follow up | | | |
|  | 3 months | | 6 months |
| Tumor volume | 100.8 cc | | Dead |
| NPRS | 0 | | 0 |
| ECOG PS | 1 | | 1 |
| Dead caused by Multiorgan failure due to outfields systemic progression | | | |

| **PATIENT_03** |
| --- |


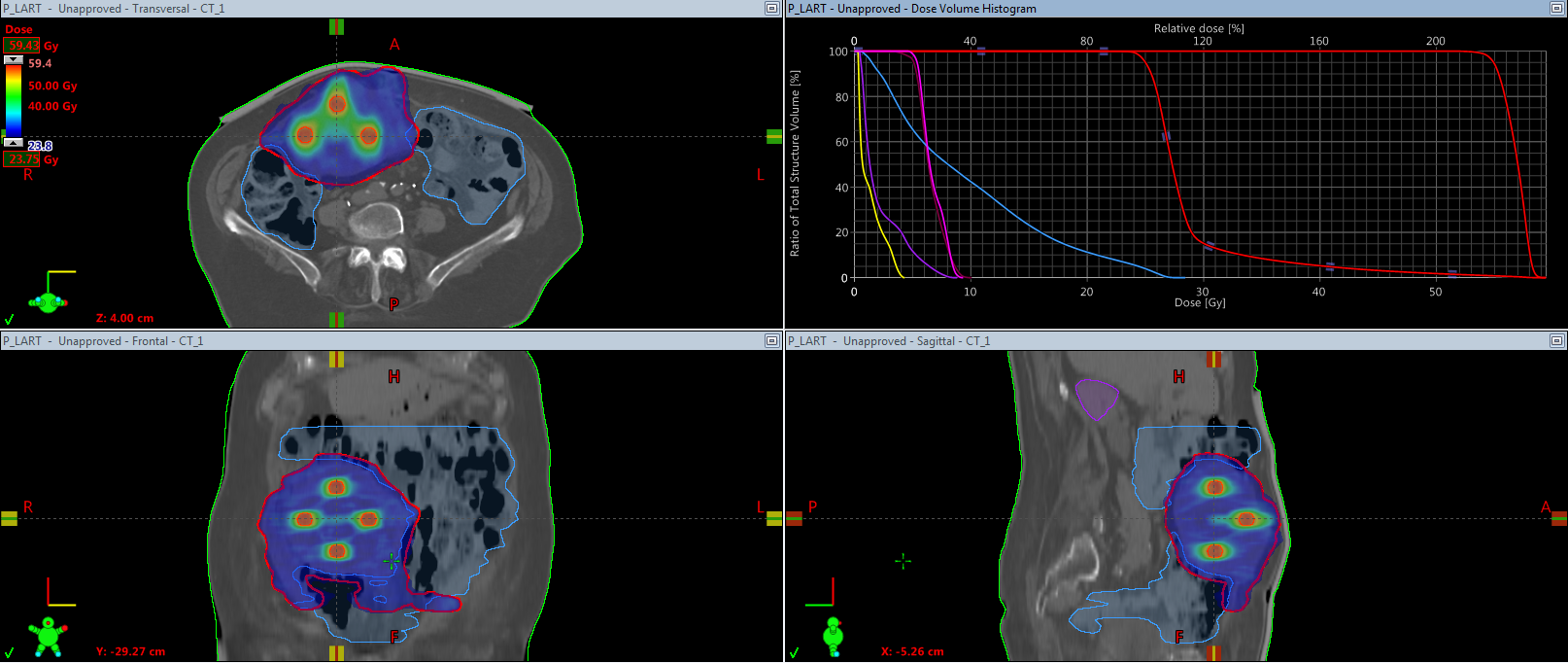


| DEMOGRAPHIC | | | |
| --- | --- | --- | --- |
| Age | | 79 | |
| Sex | | Female | |
| BMI | | 30.38 (67 Kg – 145 cm) | |
| CLINICAL DATA | | | |
| ECOG PS | | 1 | |
| NPRS | | 0 | |
| Tumor max diameter | | 17 cm | |
| Primary | | Ovarian Carcinoma | |
| Tumor location | | Pelvis | |
| Stage | | IV | |
| Target lesion | | Metastasis | |
| Antalgic Therapy | | No | |
| Therapy Lines | | I line: carboplatin-taxol  II line: carboplatin-gemcitabine-bevacizumab  III line: Niraparib | |
| Current Systemic oncological therapies | | Niraparib | |
| Hospitalization in the previews month | | No | |
| Comorbidity | | Hypertension  Dyslipidemia | |
| SFRT_DETAIL | | | |
| GTV dimension | | 784 cc | |
| PTV dimension | | 1179.2 cc | |
| Vertices_number | | 7 | |
| Monitor Units | | 2542 | |
| Energy | | Photon FFF 10 MV | |
| Arcs | | 5 | |
| Beam on time | | 2.79 min | |
| SFRT RELATED TOXICIY | | | |
| G1 | | 0 | |
| G2 | | 0 | |
| G3 | | 0 | |
| G4 | | 0 | |
| FOLLOW UP | | | |
|  | 3 months | | 6 months |
| Tumor volume | 473.1 | | 200.1 |
| NPRS | 0 | | 0 |
| ECOG PS | 1 | | 1 |
|  | | | |

| **PATIENT_04** |
| --- |


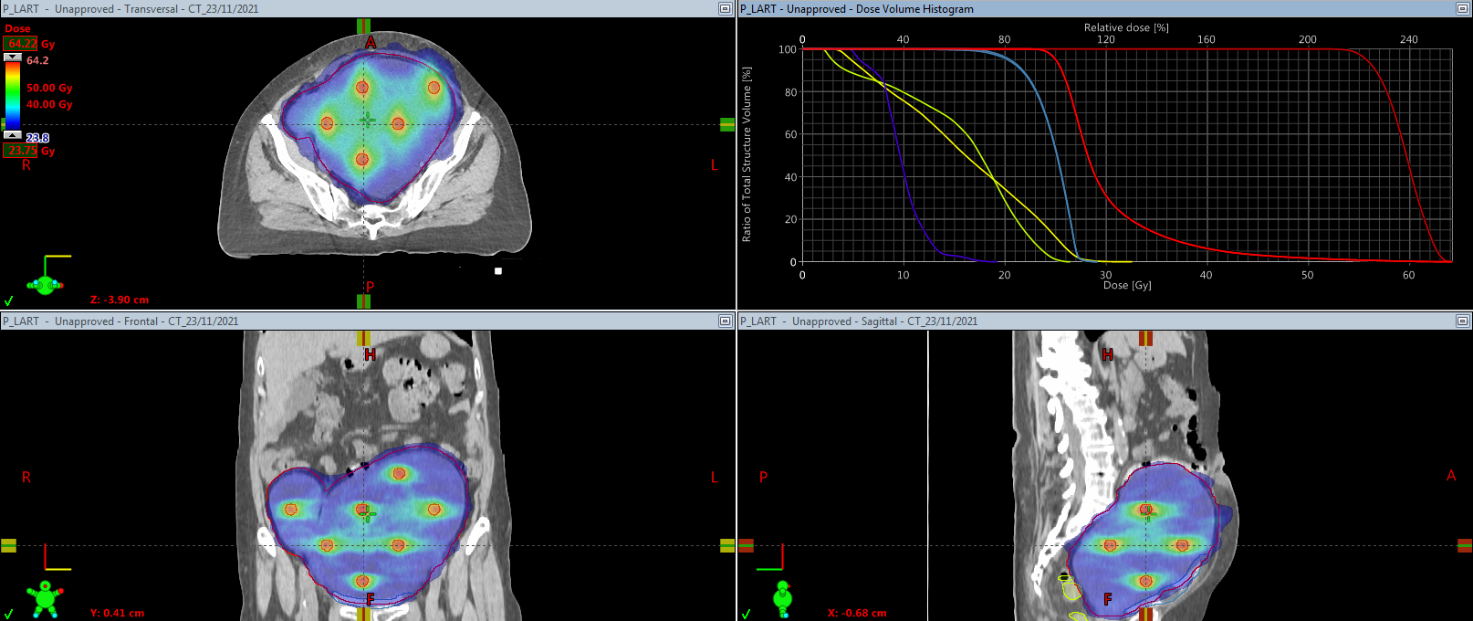


| DEMOGRAPHIC | | | |
| --- | --- | --- | --- |
| Age | | 71 | |
| Sex | | Female | |
| BMI | | 27.55 (75 kg – 165 cm) | |
| CLINICAL DATA | | | |
| ECOG PS | | 3 | |
| NPRS | | 7 | |
| Tumor max diameter | | 23 cm | |
| Primary | | Ovarian carcinoma | |
| Tumor location | | Pelvis | |
| Stage | | IV | |
| Target lesion | | Primary tumor | |
| Antalgic Therapy | | Yes | |
| Therapy Lines | | I line: carboplatin-paclitaxel  II line: vinorelbine | |
| Current Systemic oncological therapies | | Vinorelbine | |
| Hospitalization in the previews month | | Yes | |
| Comorbidity | | Hypertension  Dyslipidemia  Left hydronephrosis | |
| SFRT DETAIL | | | |
| GTV dimension | | 3838.3 | |
| PTV dimension | | 4320.3 | |
| Vertices_number | | 12 | |
| Energy | | Photon 10 MV | |
| Monitor Units | | 2427.1 | |
| Arcs | | 6 | |
| Beam on time | | 4.85 min | |
| SFRT RELATED TOXICIY | | | |
| G1 | | Fatigue | |
| G2 | | Cystitis | |
| G3 | | 0 | |
| G4 | | 0 | |
| FOLLOW UP | | | |
|  | 3 months | | 6 months |
| Tumor volume | 3504.5 cc | | 3300.5 cc |
| NPRS | 3 | | 1 |
| ECOG PS | 1 | | 1 |
|  | | | |

| **PATIENT_05** |
| --- |


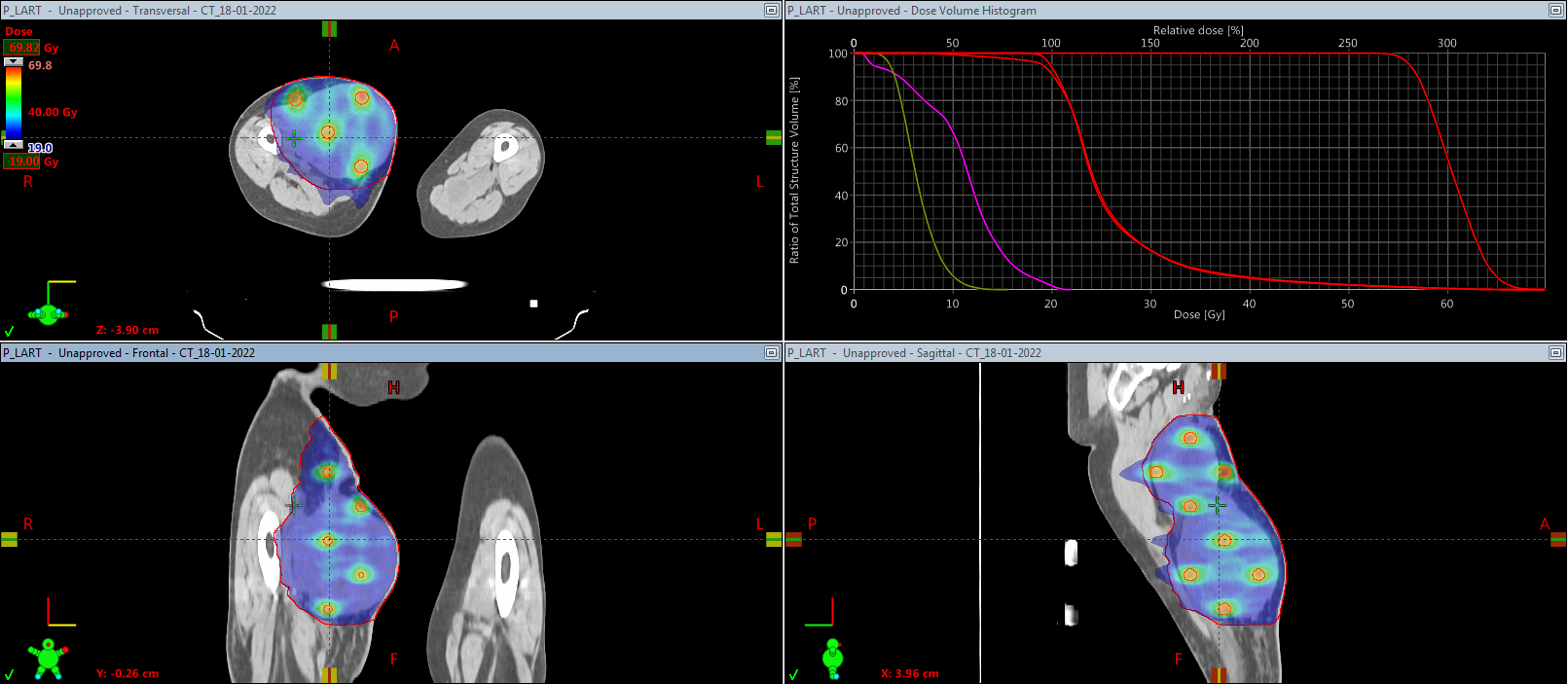


| DEMOGRAPHIC | | | |
| --- | --- | --- | --- |
| Age | | 70 | |
| Sex | | Male | |
| BMI | | 21.26 (60 kg – 168 cm) | |
| CLINICAL DATA | | | |
| ECOG PS | | 2 | |
| NPRS | | 6 | |
| Tumor max diameter | | 25 cm | |
| Primary | | Melanoma | |
| Tumor location | | Extremities (lower dx) | |
| Stage | | IV | |
| Target lesion | | Metastasis | |
| Antalgic Therapy | | Yes | |
| Therapy Lines | | I line: Nivolumab  II line Ipilumab | |
| Current Systemic oncological therapies | | Ipilumab | |
| Hospitalization in the previews month | | Yes | |
| Comorbidity | | Mitral insufficiency  Kidney failure | |
| SFRT DETAIL | | | |
| GTV dimension | | 1879.8 cc | |
| PTV dimension | | 2187 cc | |
| Vertices_number | | 13 | |
| Energy | | Photon 6 MV | |
| Monitor Units | | 2258.4 cc | |
| Arcs | | 7 | |
| Beam on time | | 4.51 min | |
| SFRT RELATED TOXICIY | | | |
| G1 | | Fatigue | |
| G2 | | 0 | |
| G3 | | 0 | |
| G4 | | 0 | |
| FOLLOW UP | | | |
|  | 3 months | | 6 months |
| Tumor volume | Dead | | Dead |
| NPRS | N/A | | N/A |
| ECOG PS | N/A | | N/A |
| Dead caused by multiorgan failure due to outfields systemic progression | | | |

| **PATIENT_06** |
| --- |


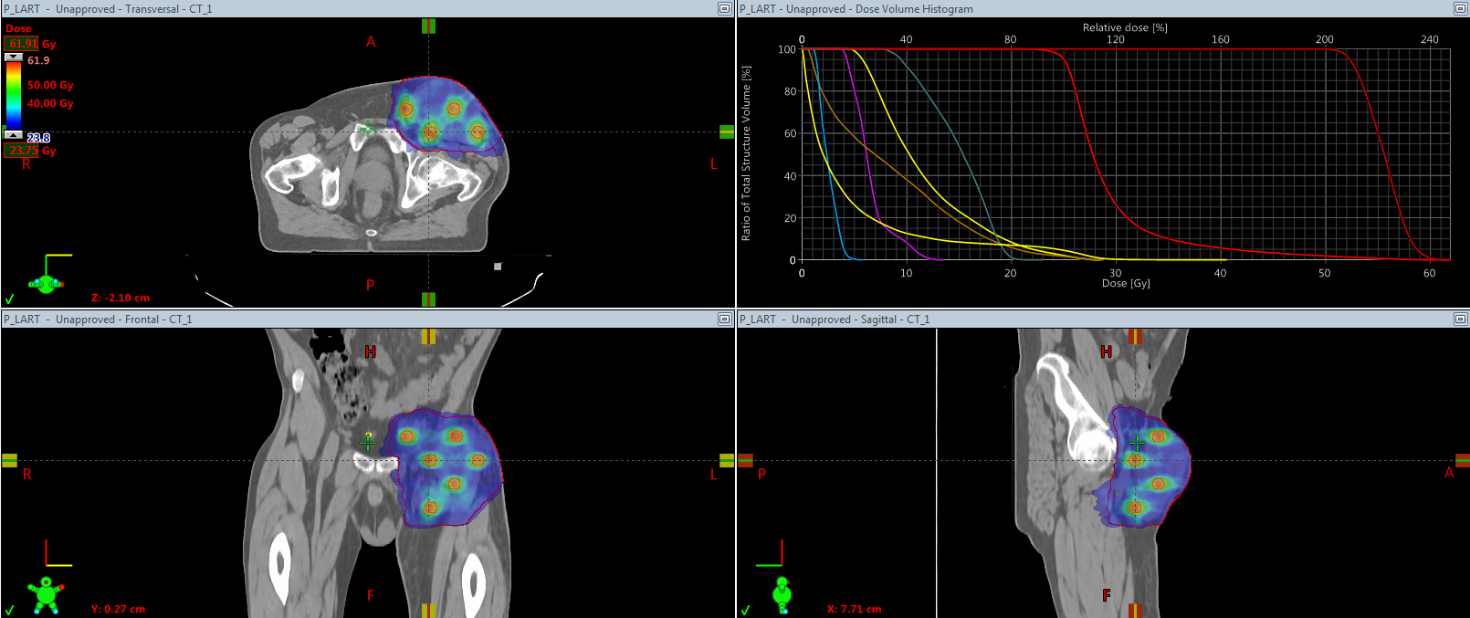


| DEMOGRAPHIC | | | |
| --- | --- | --- | --- |
| Age | | 58 | |
| Sex | | Male | |
| BMI | | 25.90 (75 kg – 170 cm) | |
| CLINICAL DATA | | | |
| ECOG PS | | 1 | |
| NPRS | | 3 | |
| Primary | | Melanoma | |
| Tumor max diameter | | 12.5 cm | |
| Tumor location | | Extremities (lower sx) | |
| Stage | | IV | |
| Target lesion | | Metastasis | |
| Antalgic Therapies | | No | |
| Therapy Lines | | I line: nivolumab  II line: ipilumab | |
| Current Systemic oncological therapies | | Ipilumab | |
| Hospitalization in the previews month | | No | |
| Comorbidity | | hyopertension  Psoriasis | |
| SFRT_DETAIL | | | |
| GTV dimension | | 908 cc | |
| PTV dimension | | 1264.3 cc | |
| Vertices_number | | 10 | |
| Energy | | Photon 10 MV | |
| Monitor Units | | 8021.1 (VMAT + IMRT) | |
| Arcs | | 8 | |
| Beam on time | | 16.04 min | |
| SFRT RELATED TOXICIY | | | |
| G1 | | 0 | |
| G2 | | 0 | |
| G3 | | 0 | |
| G4 | | 0 | |
| FOLLOW_UP | | | |
|  | 3 months | | 6 months |
| Tumor location | 309.3 cc | | 394.5 cc |
| NPRS | 0 | | 0 |
| ECOG PS | 1 | | 1 |
|  | | | |

| **PATIENT_07** |
| --- |


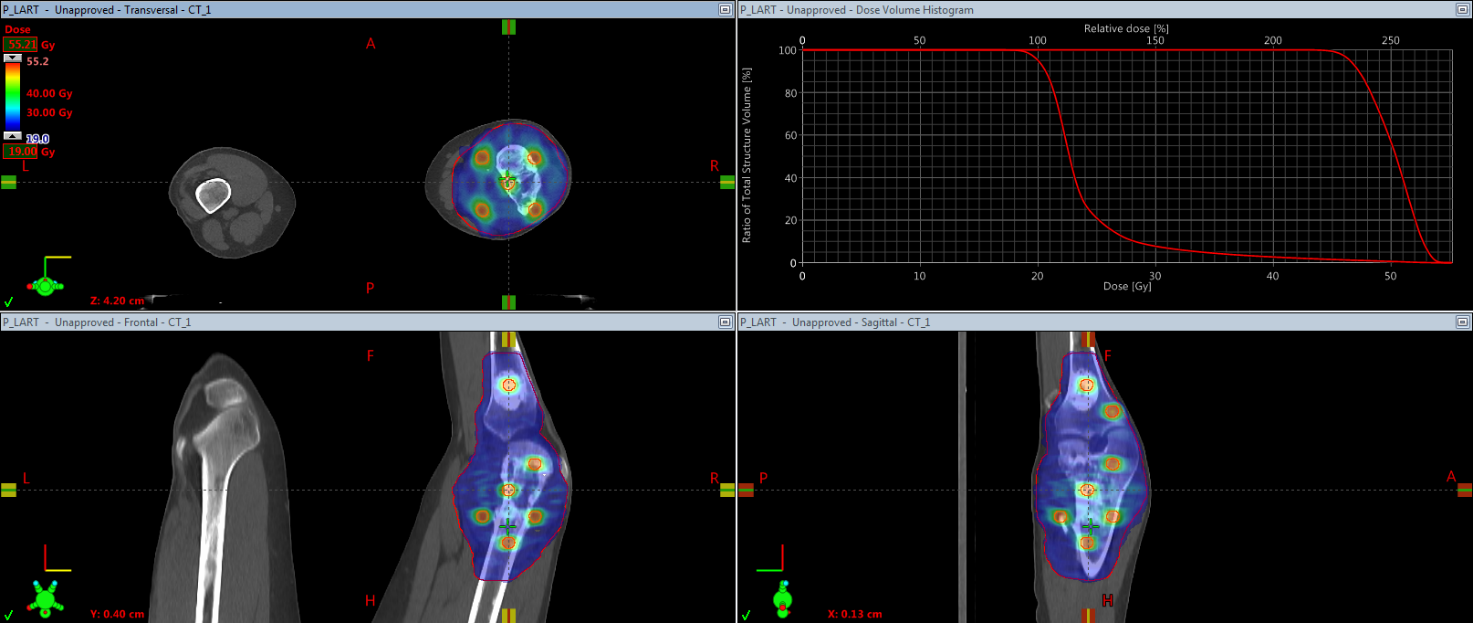


| DEMOGRAPHIC | | | |
| --- | --- | --- | --- |
| Age | | 19 years | |
| Sex | | Male | |
| BMI | | 21.91 (75 Kg – 185 cm) | |
| CLINICAL DATA | | | |
| ECOG PS | | 2 | |
| NPRS | | 10 | |
| Primary | | Sarcoma Ewing | |
| Tumor max diameter | | 27 cm | |
| Tumor location | | Extremities | |
| Stage | | IV | |
| Target lesion | | Primary Tumor | |
| Antalgic Therapy | | Yes | |
| Therapy Lines | | I line: paclitaxel  II line: temozolomide- irinotecan  III line: paclitaxel  IV line: gemcitabine | |
| Current Systemic oncological therapies | | gemcitabine | |
| Hospitalization in the previews month | | Yes | |
| Comorbidity | | 0 | |
| SFRT DETAIL | | | |
| GTV dimension | | 1517.9 cc | |
| PTV dimension | | 1755.5 cc | |
| Vertices_number | | 14 | |
| Energy | | Photon FFF 6 MV | |
| Monitor Units | | 3824.2 | |
| Arcs | | 5 | |
| Beam on time | | 3.27 min | |
| SFRT RELATED TOXICIY | | | |
| G1 | | 0 | |
| G2 | | 0 | |
| G3 | | 0 | |
| G4 | | 0 | |
| TARGET EVOLUTION | | | |
|  | 3 months | | 6 months |
| Tumor Volume | 1119.5 cc | | Dead |
| NPRS | 0 | | N/A |
| ECOG PS | 1 | | N/A |
| Dead caused by multiorgan failure due to outfields systemic progression | | | |

| **PATIENT_08** |
| --- |


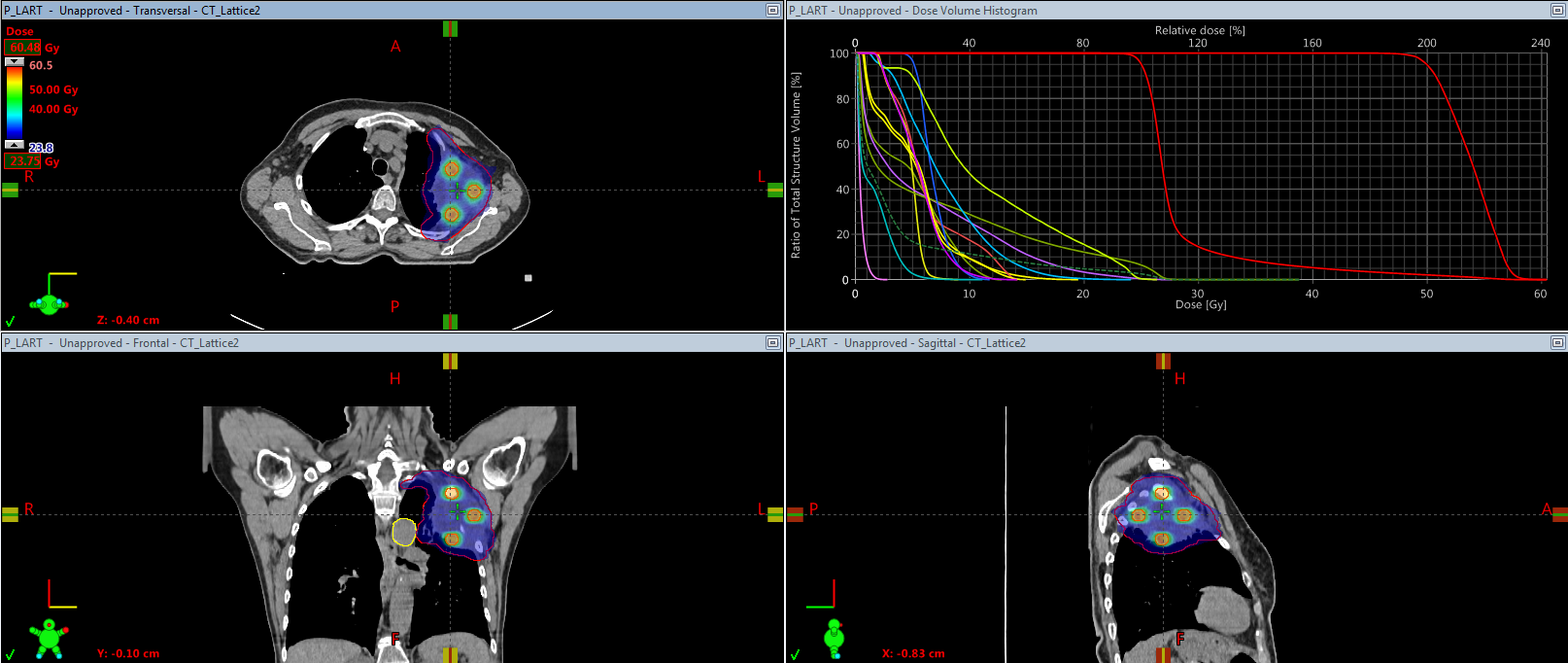


| DEMOGRAPHIC | | | |
| --- | --- | --- | --- |
| Age | | 68 years | |
| Sex | | Female | |
| BMI | | 20.03 (50 Kg – 158 cm) | |
| CLINICAL DATA | | | |
| ECOG PS | | 2 | |
| NPRS | | 8 | |
| Primary | | NSCLC | |
| Tumor max diameter | | 9 cm | |
| Tumor location | | Thorax | |
| Stage | | IV | |
| Target lesion | | Primary Lesion | |
| Antalgic Therapy | | Yes | |
| Therapy Lines | | I line: Pembrolizumab  II line: CDDP-gemcitabine  III line: Sotorasenib | |
| Current Systemic oncological therapies | | Sotorasenib | |
| Hospitalization in the previews 6 months | | No | |
| Comorbidity | | hypertension | |
| SFRT_DETAIL | | | |
| GTV dimension | | 273.2 cc | |
| PTV dimension | | 483.6 cc | |
| Vertices_number | | 6 | |
| Energy | | Photon 6 MV | |
| Monitor Units | | 2985 | |
| Arcs | | 6 | |
| Beam on time | | 6.97 min | |
| SFRT_RELATED_TOXICIY | | | |
| G1 | | 0 | |
| G2 | | 0 | |
| G3 | | 0 | |
| G4 | | 0 | |
| FOLLOW UP | | | |
|  | 3 months | | 6 months |
| Tumor volume | 82.3 cc | | dead |
| NPRS | 2 | | N/A |
| ECOG PS | 2 | | N/A |
| Dead caused by multiorgan failure due to outfields systemic progression | | | |

| **PATIENT_09** |
| --- |


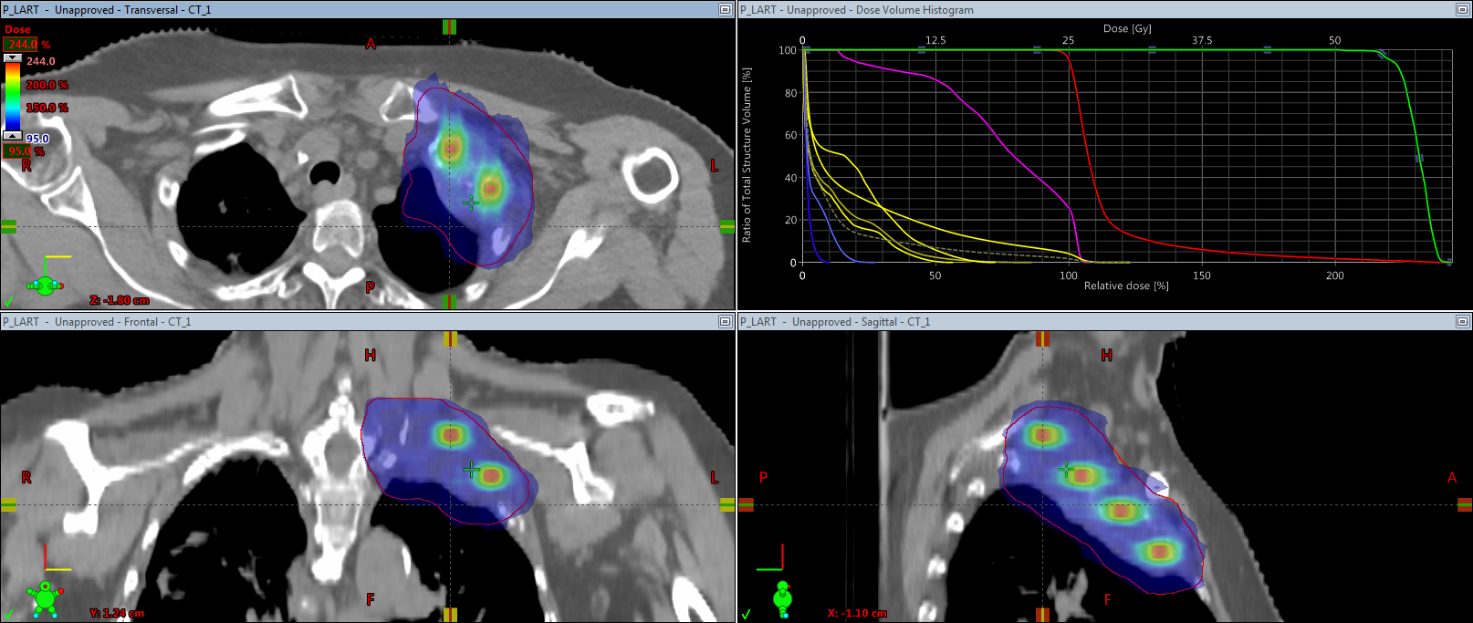


| DEMOGRAPHIC | | | |
| --- | --- | --- | --- |
| Age | | 63 | |
| Sex | | Female | |
| BMI | | 26.67 (70 kg – 162 cm) | |
| CLINICAL DATA | | | |
| ECOG PS | | 2 | |
| NPRS | | 5 | |
| Primary | | NSCLC | |
| Tumor max diameter | | 8 cm | |
| Tumor location | | Thorax | |
| Stage | | IV | |
| Target lesion | | Metastasis | |
| Antalgic Therapy | | Yes | |
| Therapy Lines | | I line: carboplatin-pemetrexed-pembrolizumab | |
| Current Systemic oncological therapies | | carboplatin-pemetrexed-pembrolizumab | |
| Hospitalization in the previews 6 months | | yes | |
| Comorbidity | | Atrial Fibrillation | |
| SFRT_DETAIL | | | |
| GTV dimension | | 168.3 cc | |
| PTV dimension | | 251.7 cc | |
| Vertices_number | | 6 | |
| Energy | | Photon 6 MV | |
| Monito Units | | 4633 | |
| Arcs | | 6 | |
| Beam on time | | 3.97 min | |
| SFRT_RELATED_TOXICIY | | | |
| G1 | | Fatigue | |
| G2 | | 0 | |
| G3 | | 0 | |
| G4 | | 0 | |
| FOLLOW UP | | | |
|  | 3 months | | 6 months |
| Tumor max diameter | 4 cm | | Dead |
| Tumor volume | 43.8 cc | | Dead |
| NPRS | 3 | | N/A |
| ECOG PS | 2 | | N/A |
| Sepsis caused by a diverticulitis | | | |

| **PATIENT_10** |
| --- |


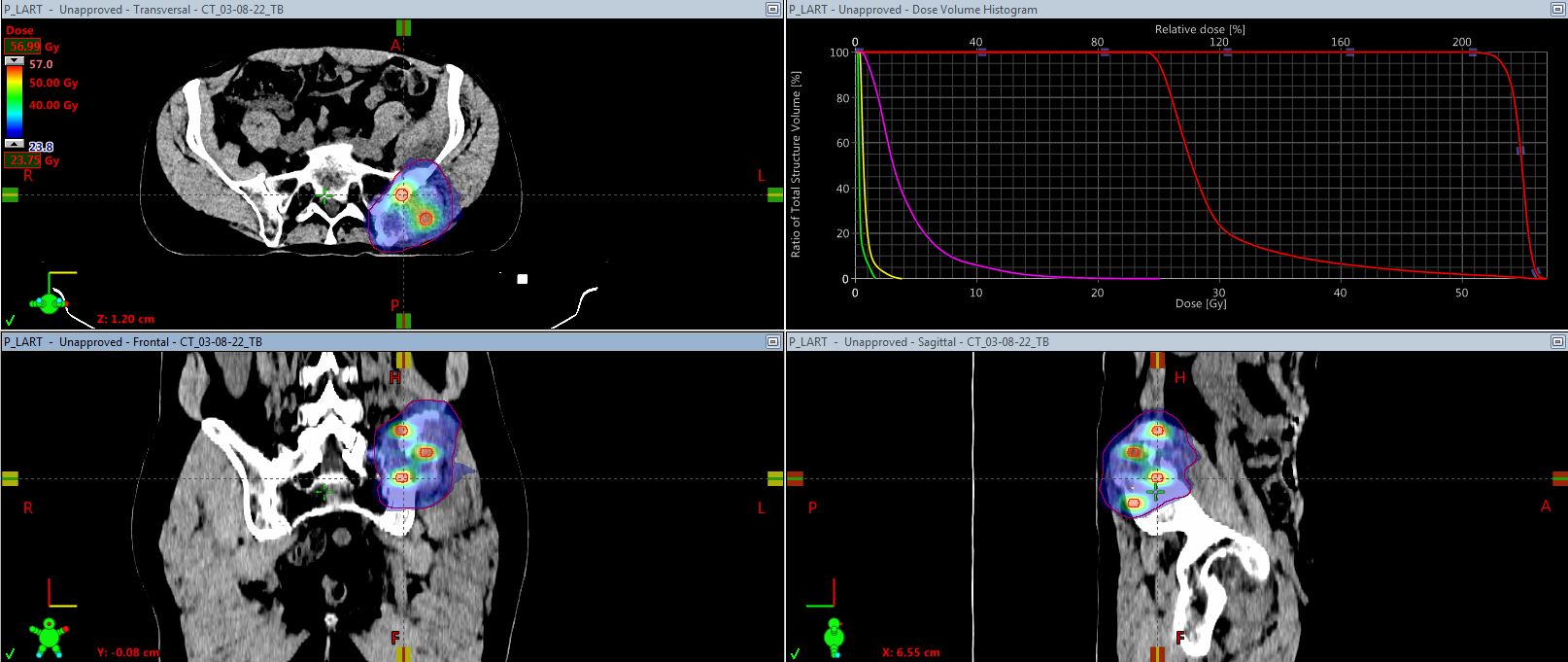


| DEMOGRAPHIC | | | |
| --- | --- | --- | --- |
| Age | | 65 years | |
| Sex | | Male | |
| BMI | | 24.57 (71 kg – 170 cm) | |
| CLINICAL DATA | | | |
| ECOG PS | | 1 | |
| NPRS | | 5 | |
| Primary | | Prostate Cancer | |
| Tumor max diameter | | 8 cm | |
| Tumor location | | Pelvis | |
| Stage | | IV | |
| Target lesion | | Metastasis | |
| Antalgic Therapy | | Yes | |
| Therapy Lines | | I line: ADT + docetaxel  II line ADT+ Enzalutamide  III line: ADT + Cabazitaxel | |
| Current Systemic oncological therapies | | ADT + Cabazitaxel | |
| Hospitalization in the previews month | | No | |
| Comorbidity | | 0 | |
| SFRT_DETAIL | | | |
| GTV dimension | | 184.3 cc | |
| PTV dimension | | 258.2 cc | |
| Vertices_number | | 6 | |
| Energy | | Photon 6 MV | |
| Monitor Units | | 2967 | |
| Arcs | | 5 | |
| Beam on time | | 5.9 min | |
| SFRT_RELATED_TOXICIY | | | |
| G1 | | Genitourinary, Fatigue | |
| G2 | | 0 | |
| G3 | | 0 | |
| G4 | | 0 | |
| FOLLOW UP | | | |
|  | 3 months | | 6 months |
| Tumor Volume | 129.6 cc | | 111.6 |
| NPRS | 0 | | 0 |
| ECOG PS | 0 | | 0 |
|  | | | |

| **PATIENT_11** |
| --- |


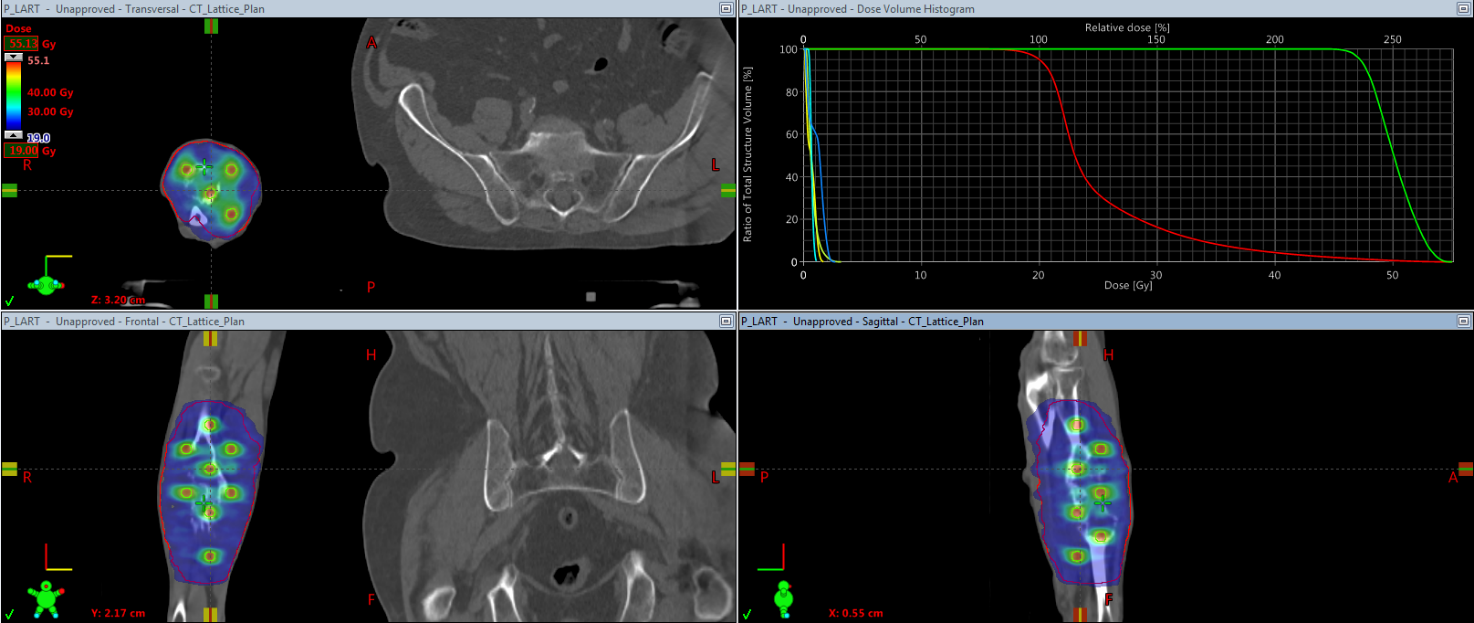


| DEMOGRAPHIC | | | |
| --- | --- | --- | --- |
| Age | | 72 | |
| Sex | | Male | |
| BMI | | 28.41 (90 kg – 178 cm) | |
| CLINICAL DATA | | | |
| ECOG PS | | 1 | |
| NPRS | | 6 | |
| Primary | | Renal Cell Carcinoma | |
| Tumor max diameter | | 17 cm | |
| Tumor location | | Extremities | |
| Stage | | IV | |
| Target lesion | | Metastasis | |
| Antalgic Therapy | | Yes | |
| Therapy Lines | | I line: Pembrolizumab-Axitinib | |
| Current Systemic oncological therapies | | Pembrolizumab-Axitinib | |
| Hospitalization in the previews month | | No | |
| Comorbidity | | COPD | |
| SFRT_DETAIL | | | |
| GTV dimension | | 479 cc | |
| PTV dimension | | 666.7 cc | |
| Vertices_number | | 17 cm | |
| Energy | | Photon 6 MV | |
| Monitor Units | | 2403.6 | |
| Arcs | | 6 | |
| Beam on time | | 4.80 | |
| SFRT_RELATED_TOXICIY | | | |
| G1 | | 0 | |
| G2 | | 0 | |
| G3 | | 0 | |
| G4 | | 0 | |
| FOLLOW UP | | | |
|  | 3 months | | 6 months |
| Tumor Volume | 134 cc | | 108.9 cc |
| NPRS | 0 | | 0 |
| ECOG PS | 0 | | 0 |
|  | | | |

| **PATIENT_12** |
| --- |


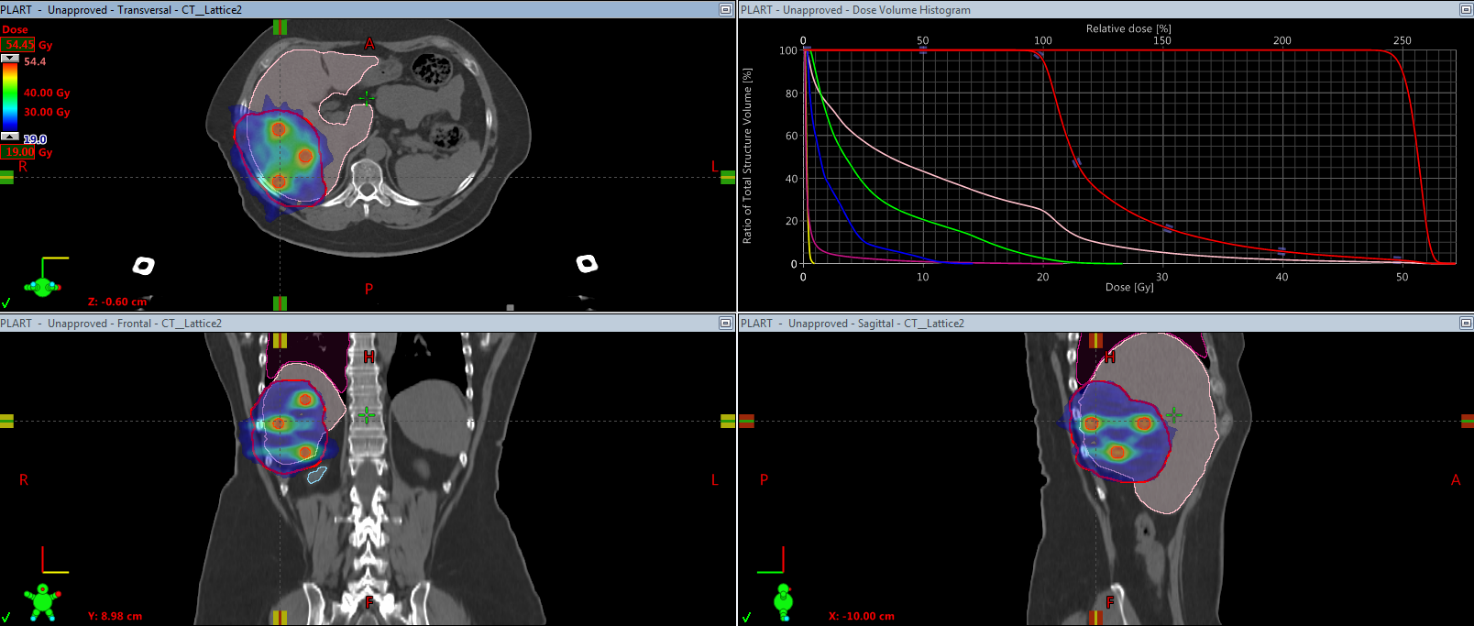


| DEMOGRAPHIC | | | |
| --- | --- | --- | --- |
| Age | | 64 years | |
| Sex | | Female | |
| BMI | | 31.64 (80 kg – 159 cm) | |
| CLINICAL DATA | | | |
| ECOG PS | | 1 | |
| NPRS | | 3 | |
| Primary | | Ovarian cancer | |
| Tumor max diameter | | 10 cm | |
| Tumor location | | Abdomen | |
| Stage | | IV | |
| Target lesion | | Metastasis | |
| Antalgic therapy | | yes | |
| Therapy Lines | | I line: carboplatin-taxol  II line: megestril  III line: carboplatin | |
| Current Systemic oncological therapies | | carboplatin | |
| Hospitalization in the previews month | | No | |
| Comorbidity | | Type II diabetes | |
| SFRT_DETAIL | | | |
| GTV dimension | | 457.8 cc | |
| PTV dimension | | 733.4 cc | |
| Vertices_number | | 7 | |
| High dose volume (V95%) | | 9.4 cc | |
| V50% | | 2083.2 cc | |
| Energy | | Photon 10 MV | |
| Monitor Units | | 3599.5 | |
| Arcs | | 4 | |
| Beam on time | | 7.1 min | |
| SFRT_RELATED_TOXICIY | | | |
| G1 | | Nausea | |
| G2 | | 0 | |
| G3 | | 0 | |
| G4 | | 0 | |
| FOLLOW UP | | | |
|  | 3 months | | 6 months |
| Tumor volume | 90.7 cc | | 32.1 cc |
| NPRS | 0 | | 0 |
| ECOG PS | 0 | | 0 |
|  | | | |

| **PATIENT_13** |
| --- |


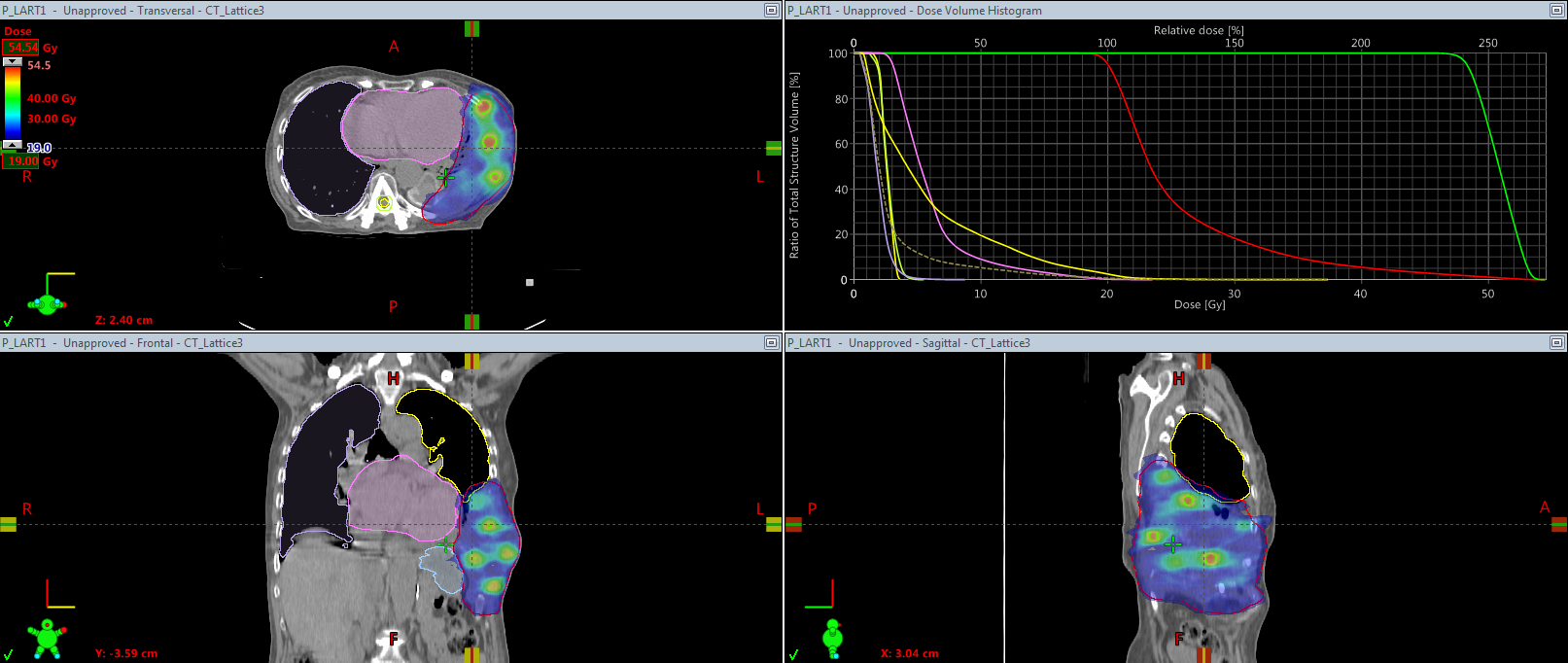


| DEMOGRAPHIC | | | |
| --- | --- | --- | --- |
| Age | | 69 years | |
| Sex | | Female | |
| BMI | | 19.95 (53 kg – 163 cm) | |
| CLINICAL DATA | | | |
| ECOG PS | | 3 | |
| NPRS | | 8 | |
| Primary | | Renal cell carcinoma | |
| Tumor max diameter | | 15 cm | |
| Tumor location | | Thorax | |
| Stage | | IV | |
| Target lesion | | Metastasis | |
| Antalgic therapy | | Yes | |
| Therapy Lines | | I line: Pembrolizumab-axitinib  II line: Sorafenib  III line: Nivolumab  IV line: Cabozantinib | |
| Current Systemic oncological therapies | | Cabozantinib | |
| Hospitalization in the previews month | | Yes | |
| Comorbidity | | 0 | |
| SFRT DETAIL | | | |
| GTV dimension | | 929.6 cc | |
| PTV dimension | | 1329. 9 cc | |
| Vertices_number | | 14 | |
| Energy | | Photon 6 MV | |
| Monitor Units | | 2477.1 | |
| Arcs | | 6 | |
| Beam on time | | 4.9 min | |
| SFRT RELATED TOXICIY | | | |
| G1 | | 0 | |
| G2 | | 0 | |
| G3 | | 0 | |
| G4 | | 0 | |
| FOLLOW UP | | | |
|  | 3 months | | 6 months |
| Tumor Volume | Dead | | dead |
| NPRS | N/A | | N/A |
| ECOG PS | N/A | | N/A |
| Dead caused by multiorgan failure due to outfields systemic progression | | | |

| **PATIENT_14** |
| --- |


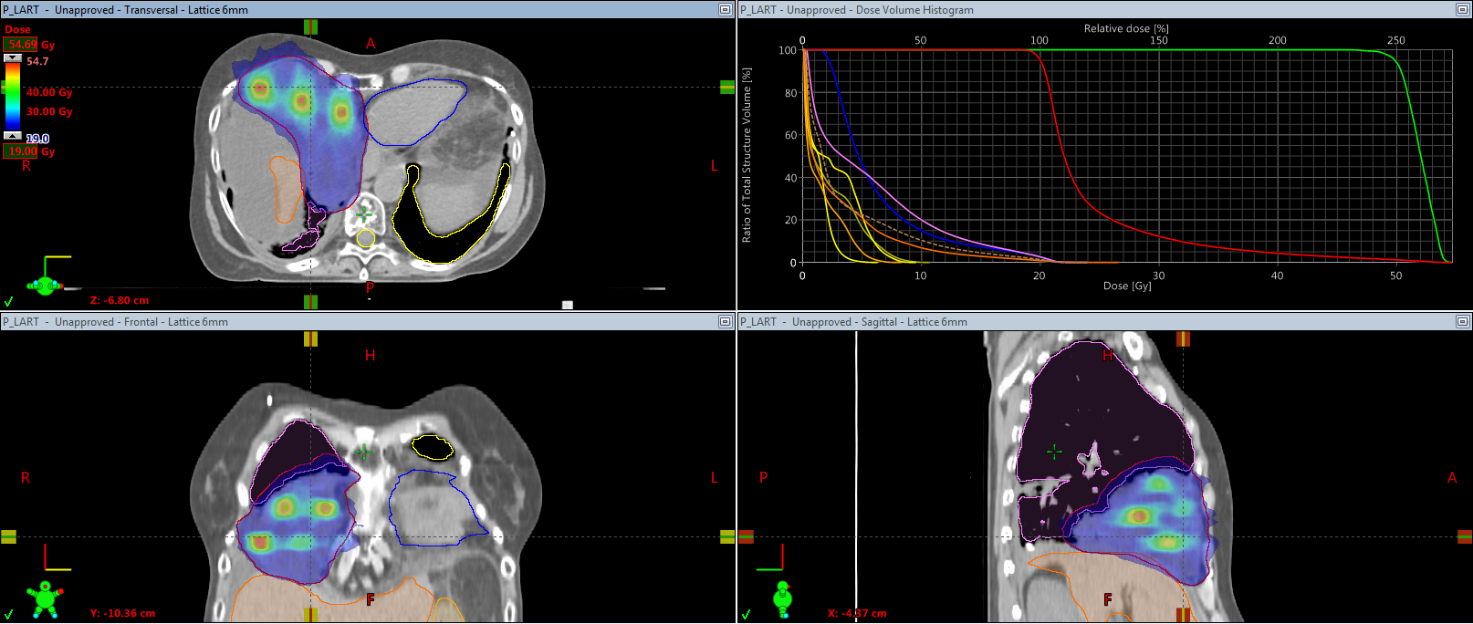


| DEMOGRAPHIC | | | |
| --- | --- | --- | --- |
| Age | | 57 | |
| Sex | | Female | |
| BMI | | 23.12 (57 kg – 157 cm) | |
| CLINICAL DATA | | | |
| ECOG PS | | 2 | |
| NPRS | | 5 | |
| Primary | | Ewing Sarcoma | |
| Tumor max diameter | | 9 cm | |
| Tumor location | | Thorax | |
| Stage | | IV | |
| Target lesion | | metastasis | |
| Antalgic Therapy | | yes | |
| Therapy Lines | | I line: ifosfamide  II line: topotecan- cyclophosphamide | |
| Current Systemic oncological therapies | | topotecan- cyclophosphamide | |
| Hospitalization in the previews month | | Yes | |
| Comorbidity | | Mitral valve disease  Uterine leiomyoma  Femur fracture | |
| SFRT_DETAIL | | | |
| GTV dimension | | 305.5 cc | |
| PTV dimension | | 480.8 cc | |
| Vertices_number | | 7 | |
| Energy | | Photon 6 MV | |
| Monitor Units | | 3026 | |
| Arcs | | 6 | |
| Beam on time | | 6 min | |
| SFRT_RELATED_TOXICIY | | | |
| G1 | | Fatigue | |
| G2 | | 0 | |
| G3 | | 0 | |
| G4 | | 0 | |
| FOLLOW UP | | | |
|  | 3 months | | 6 months |
| Tumor volume | 157 cc | | 49 cc |
| NPRS | 2 | | 1 |
| ECOG PS | 1 | | 1 |
|  | | | |

| **PATIENT_15** |
| --- |


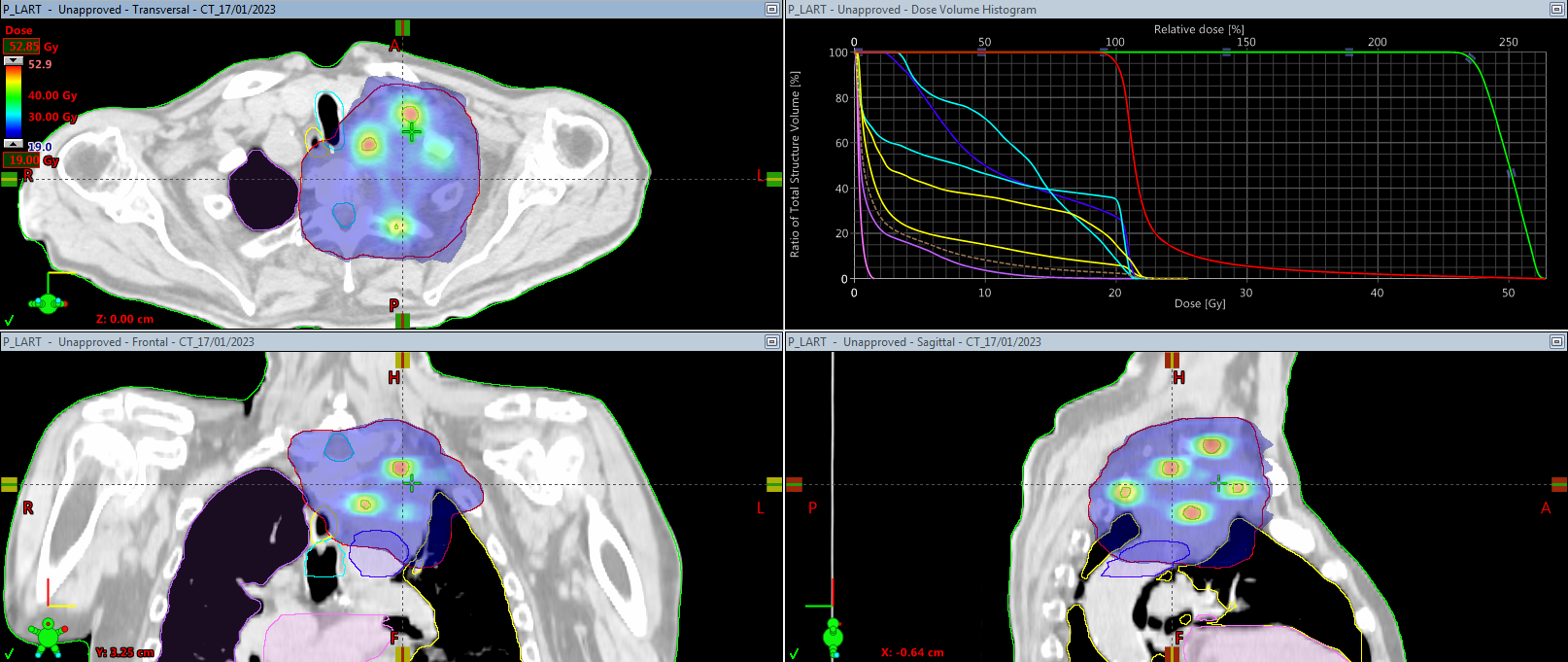


| DEMOGRAPHIC | | | |
| --- | --- | --- | --- |
| Age | | 72 | |
| Sex | | Male | |
| BMI | | 30.74 (92 kg-173 cm) | |
| CLINICAL DATA | | | |
| ECOG PS | | 3 | |
| NPRS | | 6 | |
| Primary | | NSCLC | |
| Tumor max diameter | | 12 cm | |
| Tumor location | | Thorax | |
| Stage | | IV | |
| Target lesion | | Primary lesion | |
| Antalgic therapies | | Yes | |
| Therapy Lines | | 0 | |
| Current Systemic oncological therapies | | 0 | |
| Hospitalization in the previews month | | Yes | |
| Comorbidity | | hypertension  Type II diabetes  Transient Ischemic Attack | |
| SFRT_DETAIL | | | |
| GTV dimension | | 471.7 cc | |
| PTV dimension | | 859.3 cc | |
| Vertices_number | | 8 | |
| Energy | | Photon 6 MV | |
| Monitor Units | | 4970 | |
| Arcs | | 4 | |
| Beam on time | | 10 min | |
| SFRT RELATED TOXICIY | | | |
| G1 | | 0 | |
| G2 | | 0 | |
| G3 | | 0 | |
| G4 | | 0 | |
| FOLLOW UP | | | |
|  | 3 months | | 6 months |
| Tumor volume | Dead | | dead |
| NPRS | N/A | | N/A |
| ECOG PS | N/A | | N/A |
| Dead caused by multiorgan failure due to outfields systemic progression | | | |

| **PATIENT_16** |
| --- |


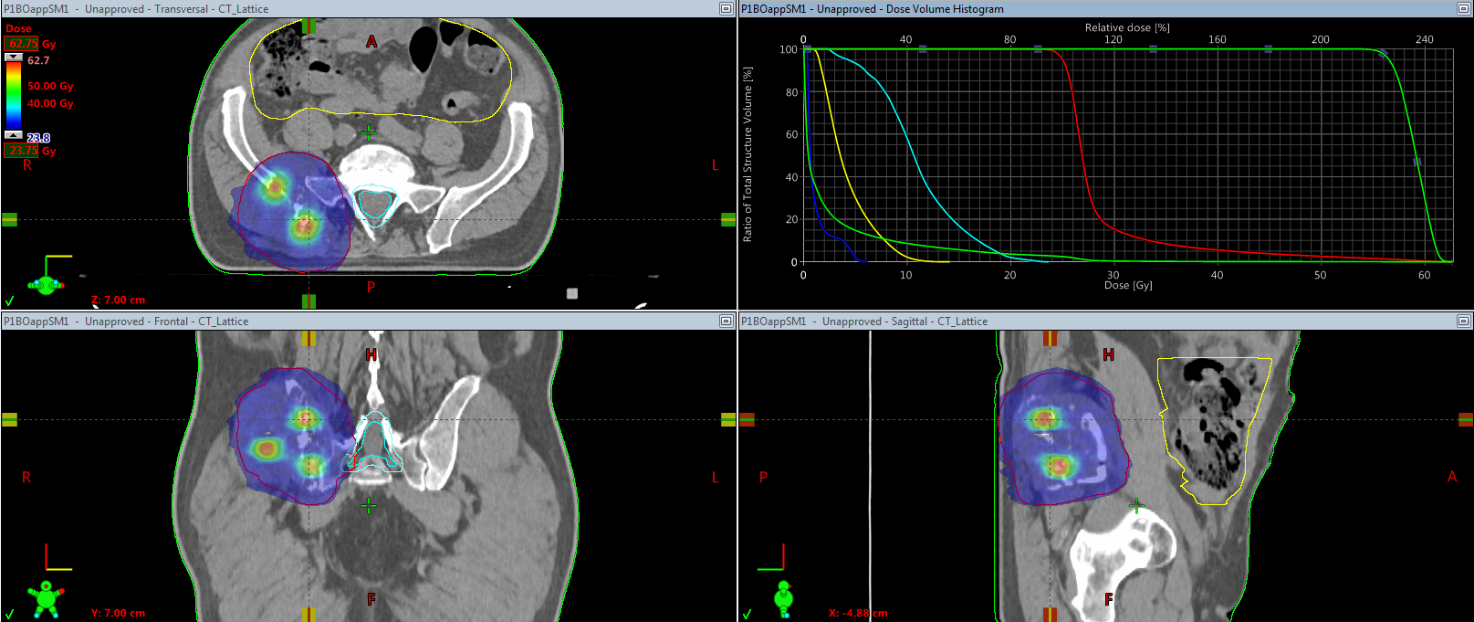


| DEMOGRAPHIC | | | |
| --- | --- | --- | --- |
| Age | | 69 | |
| Sex | | Male | |
| BMI | | 26.61 (76 kg – 169 cm) | |
| CLINICAL DATA | | | |
| ECOG PS | | 1 | |
| NPRS | | 6 | |
| Primary | | Renal cell carcinoma | |
| Tumor max diameter | | 9.5 cm | |
| Tumor location | | Pelvis | |
| Stage | | IV | |
| Target lesion | | Metastasis | |
| Antalgic Therapy | | Yes | |
| Therapy Lines | | I line: pembrolizumab-axitinib | |
| Current Systemic oncological therapies | | pembrolizumab-axitinib | |
| Hospitalization in the previews month | | yes | |
| Comorbidity | | Hypertension  Diabetes  Post-infarction ischemic heart disease (AMI) | |
| SFRT_DETAIL | | | |
| GTV dimension | | 259.5 cc | |
| PTV dimension | | 554.2 cc | |
| Vertices_number | | 5 | |
| Energy | | Photon FFF 6 MV | |
| Monitor Units | | 3380 | |
| Arcs | | 6 | |
| Beam on time | | 2.9 min | |
| SFRT_RELATED_TOXICIY | | | |
| G1 | | Fatigue | |
| G2 | | 0 | |
| G3 | | 0 | |
| G4 | | 0 | |
| FOLLOW UP | | | |
|  | 3 months | | 6 months |
| Tumor Volume | 121.7 cc | | 115.3 cc |
| NPRS | 2 | | 0 |
| ECOG PS | 0 | | 0 |
|  | | | |

| **PATIENT_17** |
| --- |


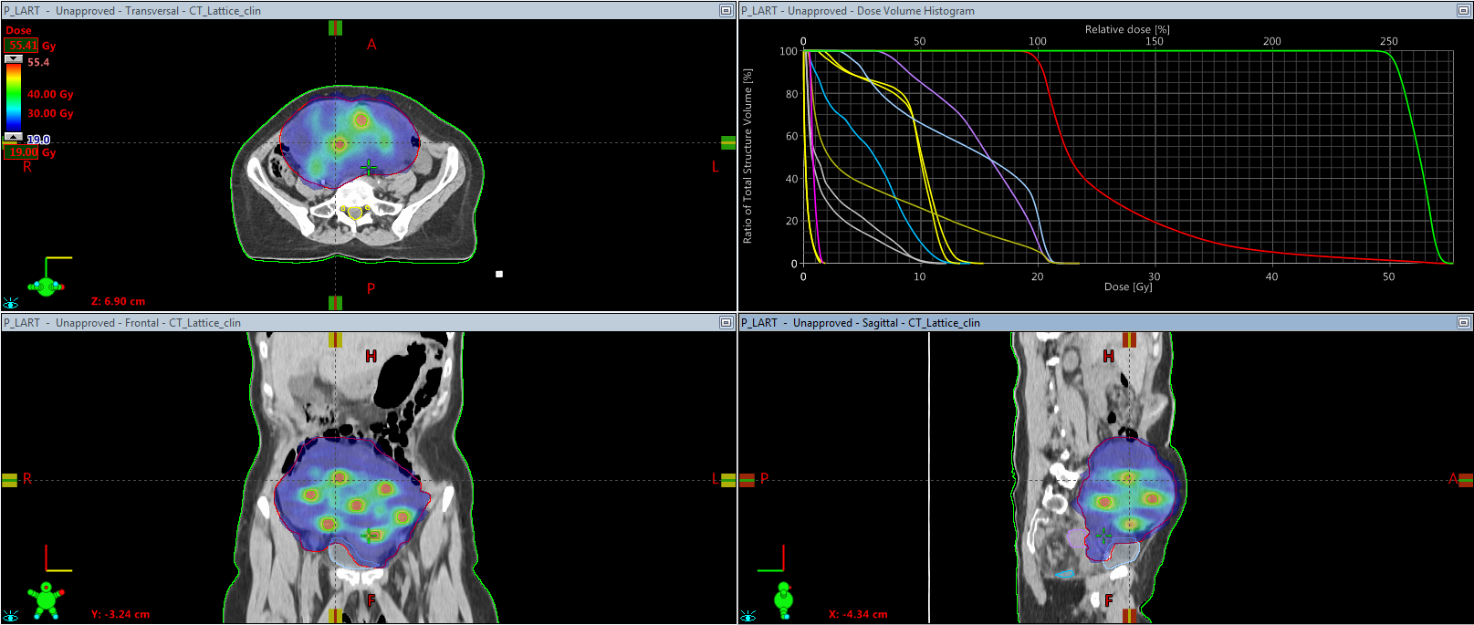


| DEMOGRAPHIC | | | |
| --- | --- | --- | --- |
| Age | | 63 | |
| Sex | | Female | |
| BMI | | 21.87 (56 kg – 160 cm) | |
| CLINICAL DATA | | | |
| ECOG PS | | 0 | |
| NPRS | | 2 | |
| Tumor max diameter | | 17 cm | |
| Primary | | Colorectal Cancer | |
| Tumor location | | Abdomen | |
| Stage | | IV | |
| Traget lesion | | Metastasis | |
| Antalgic Therapy | | Yes | |
| Therapy Lines | | I line: folfox-cetuximab  II line: bevacizumab-folfiri  III line: TAS 102 | |
| Current Systemic oncological therapies | | TAS 102 | |
| Hospitalization in the previews month | | No | |
| Comorbidity | | Appendixotomy | |
| SFRT_DETAIL | | | |
| GTV dimension | | 1150.3 cc | |
| PTV dimension | | 1685.1 cc | |
| Vertices_number | | 14 | |
| Energy | | Photon 10 MV | |
| Monitor Units | | 3956.5 | |
| Arcs | | 6 | |
| Beam on time | | 8 min | |
| SFRT_RELATED_TOXICIY | | | |
| G1 | | Fatigue | |
| G2 | | Genitourinary | |
| G3 | | 0 | |
| G4 | | 0 | |
| FOLLOW UP | | | |
| Tumor volume | 16 cm | | 15.5 cm |
| GTV | 1077.9 cc | | 944.3 cc |
| NPRS | 0 | | 0 |
| ECOG PS | 0 | | 0 |
|  | | | |

| **PATIENT_18** |
| --- |


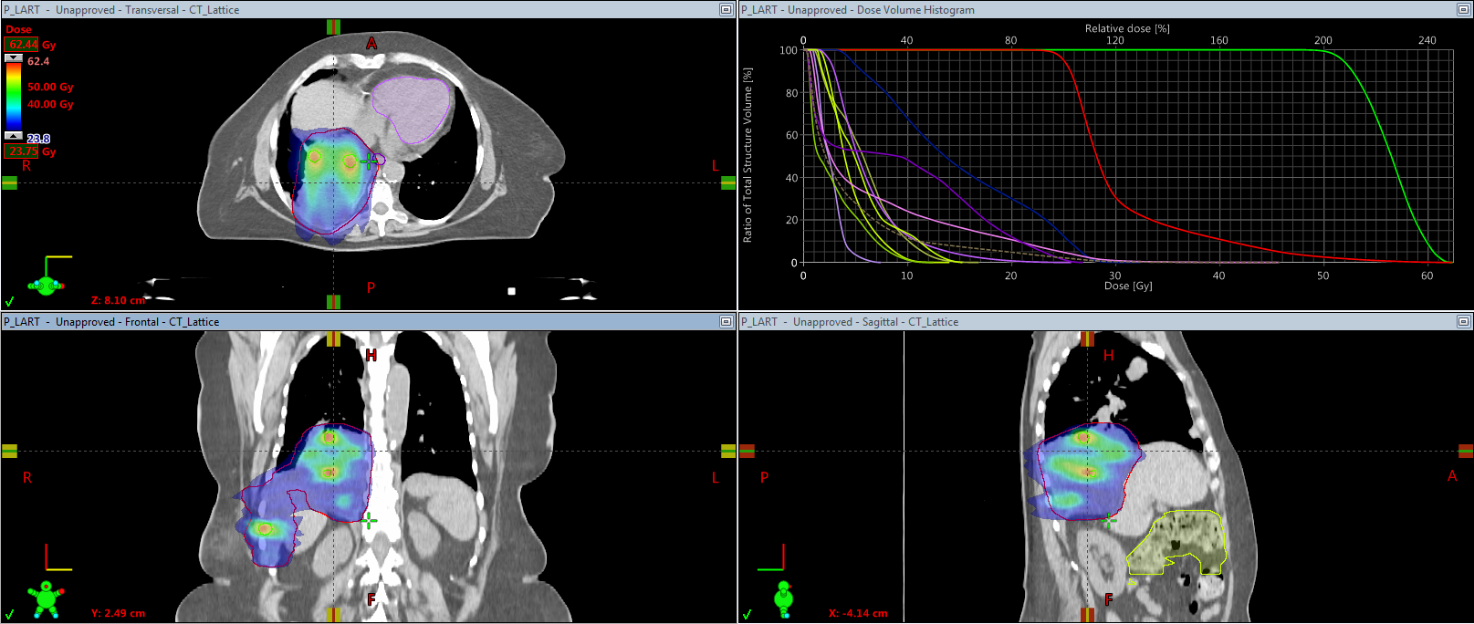


| DEMOGRAPHIC | | | |
| --- | --- | --- | --- |
| Age | | 83 | |
| Sex | | Female | |
| BMI | | 30.04 (75 kg – 158 cm) | |
| CLINICAL DATA | | | |
| ECOG PS | | 2 | |
| NPRS | | 8 | |
| Primary | | Mesothelioma | |
| Tumor max diameter | | 18 cm | |
| Tumor location | | Thorax | |
| Stage | | IV | |
| Target lesion | | Primary lesion | |
| Antalgic Therapy | | Yes | |
| Therapy Lines | | I line: gemcitabina | |
| Current Systemic oncological therapies | | gemcitabina | |
| Hospitalization in the previews month | | Yes | |
| Comorbidity | | Type II Diabetes  Ischemic stroke  Hypertension | |
| SFRT DETAIL | | | |
| GTV dimension | | 784.2 cc | |
| PTV dimension | | 1194.2 cc | |
| Vertices_number | | 8 | |
| Energy | | Photon 6 MV | |
| Monitor Units | | 2098.2 | |
| Arcs | | 4 | |
| Beam on time | | 4 min | |
| SFRT RELATED TOXICIY | | | |
| G1 | | Esophagitis | |
| G2 | | Fatigue | |
| G3 | | 0 | |
| G4 | | 0 | |
| FOLLOW UP | | | |
| Baseline | 3 months | | 6 months |
| Tumor Volume | dead | | dead |
| NPRS | N/A | | N/A |
| ECOG PS | N/A | | N/A |
| Dead caused by multiorgan failure due to outfields systemic progression | | | |

| **PATIENT_19** |
| --- |


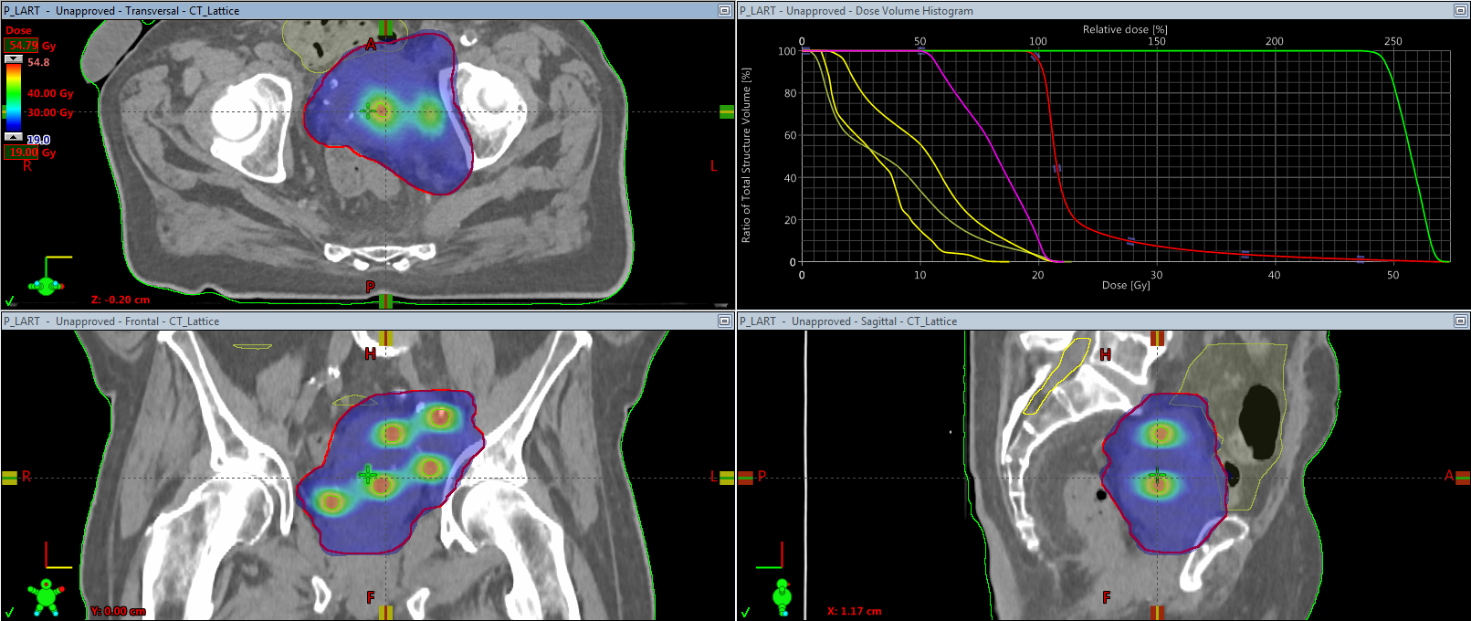


| DEMOGRAPHIC | | | |
| --- | --- | --- | --- |
| Age | | 73 | |
| Sex | | Male | |
| BMI | | 30.42 (90 kg - 172 cm) | |
| CLINICAL DATA | | | |
| ECOG PS | | 2 | |
| NPRS | | 8 | |
| Primary | | Bladder Cancer | |
| Tumor max diameter | | 12 cm | |
| Tumor location | | Pelvis | |
| Stage | | IV | |
| Target location | | Metastasis | |
| Antalgic therapy | | Yes | |
| Therapy Lines | | 0 | |
| Current Systemic oncological therapies | | 0 | |
| Hospitalization in the previews month | | yes | |
| Comorbidity | | Hypertension  Type II Diabetes  Dyslipidemia | |
| SFRT DETAIL | | | |
| GTV dimension | | 333.2 cc | |
| PTV dimension | | 561.4 cc | |
| Vertices_number | | 5 | |
| Energy | | Photon 10 MV | |
| Monito Units | | 3522.1 | |
| Arcs | | 4 | |
| Beam on time | | 7 min | |
| SFRT RELATED TOXICIY | | | |
| G1 | |  | |
| G2 | | Proctitis- fatigue | |
| G3 | |  | |
| G4 | |  | |
| FOLLOW UP | | | |
|  | 3 months | | 6 months |
| Tumor volume | dead | | Dead |
| NPRS | N/A | | N/A |
| ECOG PS | N/A | | N/A |
| Dead caused by multiorgan failure due to outfields systemic progression | | | |

| **PATIENT_20** |
| --- |


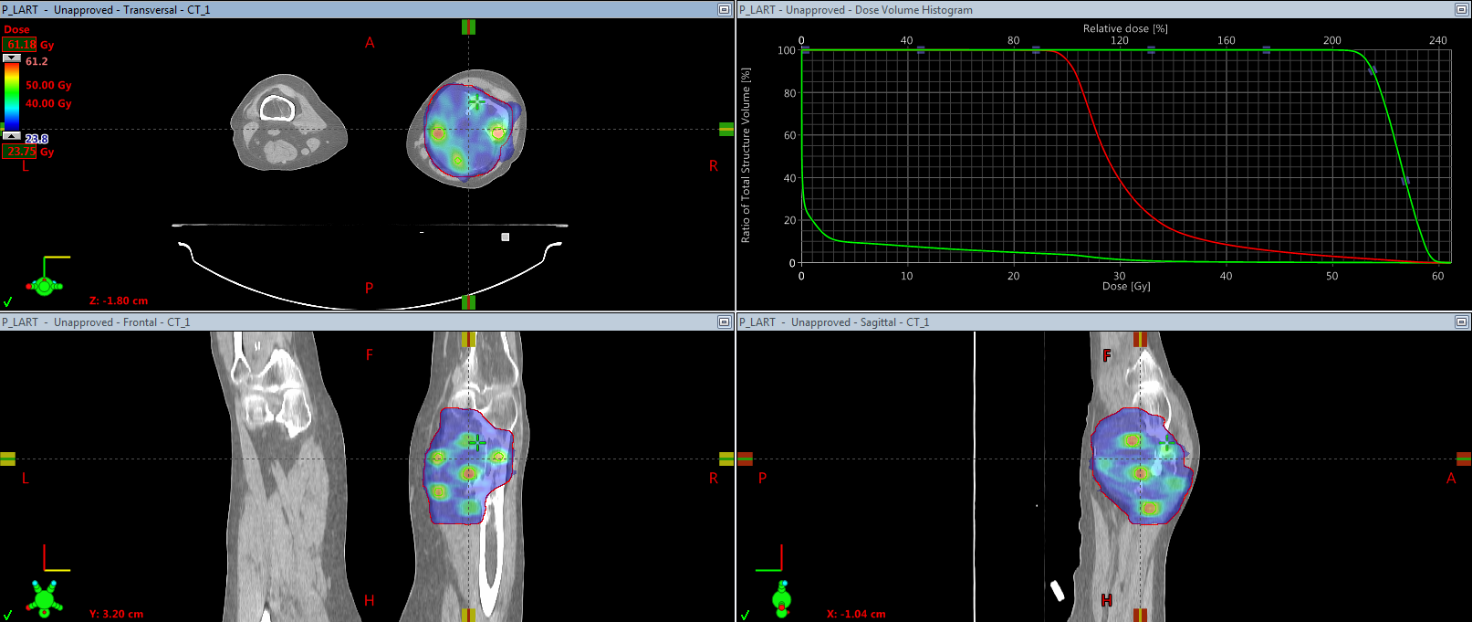


| DEMOGRAPHIC | | | |
| --- | --- | --- | --- |
| Age | | 70 | |
| Sex | | Female | |
| BMI | | 25.71 (70 kg – 165 cm) | |
| CLINICAL DATA | | | |
| ECOG PS | | 2 | |
| NPRS | | 7 | |
| Primary | | NSCLC | |
| Tumor max diameter | | 13 cm | |
| Tumor location | | Extremities | |
| Stage | | IV | |
| Target lesion | | Metastasis | |
| Antalgic Therapy | | Yes | |
| Therapy Lines | | I line: pembrolizumab-pemetrexed-carboplatin | |
| Current Systemic oncological therapies | | pembrolizumab | |
| Hospitalization in the previews 6 months | | Yes | |
| Comorbidity | | Hypothyroidism  hypertension  COPD  Acute myocardial infarction | |
| SFRT DETAIL | | | |
| GTV dimension | | 641.8 cc | |
| PTV dimension | | 888.2 cc | |
| Vertices_number | | 10 | |
| Energy | | Photon 6 MV | |
| Monitor Units | | 3342.8 | |
| Arcs | | 5 | |
| Beam on time | | 7 min | |
| SFRT RELATED TOXICIY | | | |
| G1 | | 0 | |
| G2 | | Fatigue | |
| G3 | | 0 | |
| G4 | | 0 | |
| FOLLOW UP | | | |
|  | 3 months | | 6 months |
| Tumor volume | dead | | dead |
| NPRS | N/A | | N/A |
| ECOG PS | N/A | | N/A |
